# Efficient Spin-Flip between Charge-Transfer States for High-Performance Electroluminescence, without an Intermediate Locally Excited State

**DOI:** 10.34133/research.0155

**Published:** 2023-05-25

**Authors:** Dong-Hai Zhang, Shanshan Jiang, Xiaodong Tao, Fulin Lin, Lingyi Meng, Xu-Lin Chen, Can-Zhong Lu

**Affiliations:** ^1^ State Key Laboratory of Structural Chemistry, Fujian Institute of Research on the Structure of Matter, Chinese Academy of Sciences, Fuzhou, Fujian 350002, China.; ^2^ Fujian Science and Technology Innovation Laboratory for Optoelectronic Information of China, Fuzhou, Fujian 350108, China.; ^3^ Xiamen Key Laboratory of Rare Earth Photoelectric Functional Materials, Xiamen Institute of Rare Earth Materials, Haixi Institutes, Chinese Academy of Sciences, Xiamen, Fujian 361021, China.; ^4^ University of Chinese Academy of Sciences, Beijing 100049, China.

## Abstract

Thermally activated delayed fluorescence (TADF) materials with both high photoluminescence quantum yield (PLQY) and fast reverse intersystem crossing (RISC) are strongly desired to realize efficient and stable organic light-emitting diodes (OLEDs). Control of excited-state dynamics via molecular design plays a central role in optimizing the PLQY and RISC rate of TADF materials but remains challenging. Here, 3 TADF emitters possessing similar molecular structures, similar high PLQYs (89.5% to 96.3%), and approximate energy levels of the lowest excited singlet states (S_1_), but significantly different spin-flipping RISC rates (0.03 × 10^6^ s^−1^ vs. 2.26 × 10^6^ s^−1^) and exciton lifetime (297.1 to 332.8 μs vs. 6.0 μs) were systematically synthesized to deeply investigate the feasibility of spin-flip between charge-transfer excited states (^3^CT–^1^CT) transition. Experimental and theoretical studies reveal that the small singlet–triplet energy gap together with low RISC reorganization energy between the ^3^CT and ^1^CT states could provide an efficient RISC through fast spin-flip ^3^CT–^1^CT transition, without the participation of an intermediate locally excited state, which has previously been recognized as being necessary for realizing fast RISC. Finally, the OLED based on the champion TADF emitter achieves a maximum external quantum efficiency of 27.1%, a tiny efficiency roll-off of 4.1% at 1,000 cd/m^2^, and a high luminance of 28,150 cd/m^2^, which are markedly superior to those of the OLEDs employing the other 2 TADF emitters.

## Introduction

Organic molecules featuring thermally activated delayed fluorescence (TADF) have emerged as one of the most attractive emitters for the realization of high-efficiency organic light-emitting diodes (OLEDs), owing to their potential utilization of the electro-generated triplet excitons via reverse intersystem crossing (RISC) [1]. Nevertheless, challenges remain for TADF-based OLEDs to become truly practical and commercialized [2,3]. One outstanding challenge is severe efficiency roll-off under high current densities, which stem from the exciton annihilation processes and formation of high-energy excitons in the emitting layer [4–12]. The key solution to this issue lies in developing TADF emitters with fast RISC processes and short excited-state lifetimes [13–18]. At room temperature, in general, RISC involving spin-flip and up-conversion from the lowest triplet states (T_1_) to the lowest excited singlet states (S_1_) is a rate-determining step in TADF process [19,20]. According to first-order perturbation theory, a small energy gap (Δ*E*_ST_) and a large spin-orbit coupling (SOC) value between S_1_ and T_1_ are required for efficient RISC [21]. To attain a small Δ*E*_ST_, TADF molecules are generally designed with highly twisted or separated electron donor (D) and acceptor (A) chromophores, leading to spatially separated frontier molecular orbitals (FMOs) and radiative charge transfer (CT) transitions [22]. In such instances, however, it is widely accepted that the SOC between S_1_ and T_1_ states that have the same CT excitation character is negligible according to the El-Sayed rule, which always leads to a conclusion that the RISC rates are very limited in these TADF systems [23,24]. In this context, a 3-state model has recently been developed to rationalize the high RISC rates of some TADF materials, which ascribes the increased RISC rate between the CT states (S_1_ and T_1_) to their coupling with the energetically close locally excited triplet states (^3^LE) [25–32]. In the past years, various molecular design strategies have been proposed to introduce an intermediate ^3^LE state or partially mix it with the ^3^CT, aiming at accelerating the spin-flipping RISC process [24,29,33,34].

Despite these advances, it remains controversial and difficult to reach a definitive conclusion to account for the RISC of organic TADF molecules, or, alternatively, there exist a wide variety of TADF molecular systems to which the above-mentioned mechanisms do not apply [6,35–41]. Some investigations indicate that the El-Sayed rule has limitations, especially in inhomogeneous media. Park and coworkers demonstrated that, by taking into account the contribution of rotamers, the statistically weighted SOC between ^1^CT and ^3^CT is not zero; the spin-flips between them are rotationally or vibronically activated without the participation of ^3^LE states [42]. Furthermore, according to Marcus’ theory, RISC rate constant (*k*_RISC_) is given by:

$$k_{\mathrm{RISC}}=\frac{1}{\hbar }\left|<S_{1}\right|{\hat{H}}_{\mathrm{SOC}}{\left|T_{1}>\right|}^{2}\sqrt{\frac{\pi }{\lambda_{\mathrm{RISC}}k_{B}T}}\exp\left[-\frac{{\left(\Delta E_{\mathrm{ST}}+\lambda_{\mathrm{RISC}}\right)}^{2}}{4\lambda_{\mathrm{RISC}}k_{B}T}\right]$$ (1)

where $<S_{1}\left|{\hat{H}}_{\mathrm{SOC}}\right|T_{1}>$ is the SOC matrix element between the S_1_ and T_1_ states and Δ*E*_ST_ is their energy gap; λ_RISC_ is the reorganization energy for the RISC transition [43–47]. This relationship suggests that Δ*E*_ST_, SOC constant, and reorganization energy should be taken into overall consideration to realize efficient RISC in a TADF molecule. In practice, however, reorganization energy has rarely been considered when evaluating the RISC process [16,20,44,48]. It is intriguing and challenging to balance the trade-off between the 3 factors mentioned above and to identify the decisive factors for the RISC process of a TADF system.

We report herein 3 high-efficiency (photoluminescence quantum yield, PLQY = 89.5% to 96.3%) TADF molecules consisting of ortho-connected D and A units. Based on similar molecular structures and energy levels of S_1_, our designs focus on systematically manipulating the components of low-lying excited states via tuning the CT interaction and their effect on TADF characteristics especially RISC rate. Interestingly, fine-tuning the components of low-lying excited states leads to distinctly (over an order of magnitude) different RISC rate constants and delayed fluorescence lifetimes, although in approximate energy levels of S_1_. Enhancing the CT components of low-lying excited states stabilizes the CT excited states and narrows Δ*E*_ST_, while it has little effect on the lowest ^3^LE states (T_2_) of these molecules, which localize on the acceptor units. We demonstrate that the up-lying ^3^LE states do not participate the ISC and RISC processes due to the high energy gap of Δ*E*_S1-T2_ (~0.25 eV) and Δ*E*_T1-T2_ (0.3 to 0.4 eV); the ^3^CT–^1^CT transition provides a much higher RISC rate through fast spin-flip ^3^CT–^1^CT transition than the transition from hybridized local and charge transfer (^3^HLCT) to ^1^CT due to smaller Δ*E*_ST_ together with distinctly lower RISC reorganization energy. Eventually, the OLED employing our champion TADF emitter realized a maximum external quantum efficiency (EQE_max_) of 27.1% and a very small roll-off of 4.1% at a high luminance of 1,000 cd/m^2^.

## Results

### Molecular design, crystal structures, and theoretical calculation

**Fig. 1.**
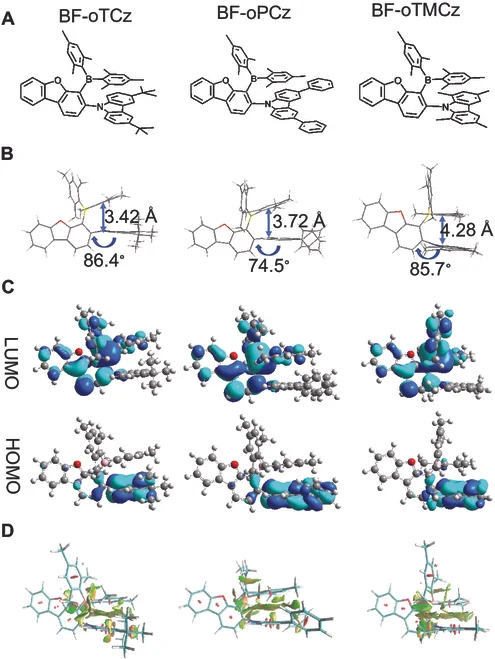
(A) Chemical structures. (B) Single-crystal structures. (C) Theoretical LUMO and HOMO distributions. (D) RDG isosurface of BF-oTCz, BF-oPCz, and BF-oTMCz.

The 3 TADF molecules, BF-oTCz, BF-oPCz, and BF-oTMCz, are designed with dimesitylborane-dibenzofuran (BF) electron-acceptors and carbazole-derived electron-donors (Fig. 1A). Bulky 1,3,5-trimethylbenzene is introduced to protect the tricoordinate boron center from being attacked by nucleophiles. The donor type is finely tuned to manipulate the components of low-lying excited states. Synthesis of each designed TADF molecule was carried out by the 2-step reaction with an overall yield surpassing 40%, as depicted in the Supplementary Materials (Scheme S1). The obtained compounds were purified by column chromatography and temperature-gradient vacuum sublimation and then were fully characterized using NMR spectroscopy and elemental analysis.

The single-crystal structures obtained from x-ray diffraction analysis are presented in Fig. 1B. For each molecule, the carbazole unit and triarylboron unit are ortho-linked on an electron-withdrawing dibenzofuran unit. The steric hindrance leads to a large dihedral angle (74.5° to 86.4°) between the carbazole and dibenzofuran units and a face-to-face D–A alignment in close proximity. The carbazole donors are nearly parallel to one of the aryl rings of the triarylboron acceptor with small distances ranging from 3.42 to 4.28 Å, indicating strong intramolecular interactions between each other, which are expected not only to confine the molecular configuration, but also to provide a through-space charge transfer (TSCT) pathway in the stacking space, in addition to through-bond charge transfer (TBCT) [49–56]. These inferences were further validated by reduced density gradient (RDG) analysis of their crystal structures. The large and nearly continuous region in RDG isosurface maps (Fig. 1D) together with the obvious spikes in the region that Sign (λ_2_) ρ is near zero in the scatter diagrams (Fig. S2) reveal the presence of significant intramolecular interactions and TSCT transitions between the close-range face-to-face D and A moieties for all these compounds. The significant D–A interactions could suppress the molecular rotational and vibrational relaxations and thus are conducive to realizing high PLQYs.

To investigate the electronic properties of these emitters, density functional theory (DFT) and time-dependent DFT (TD-DFT) calculations were performed at the PBE0/6-311g (d, p) level based on the single-crystal structures [57–59]. Due to the large torsion angles between the carbazole and dibenzofuran units, FMO distributions of these molecules are effectively separated. As shown in Fig. 1C, the highest occupied molecular orbitals (HOMOs) are mainly localized on the carbazole donor moieties and slightly extended to the dibenzofuran units, and the lowest unoccupied molecular orbitals (LUMOs) are entirely distributed over the dimesitylboron and dibenzofuran units. A small overlap of FMOs always gives rise to S_1_ with CT character and also leads to small ^1^CT–^3^CT splitting, which is a necessary but not sufficient condition for realizing small Δ*E*_ST_ and efficient RISC. The TD-DFT calculations (Fig. S3) reveal that the S_1_ states of these 3 molecules show predominant CT contributions ranging from 86.3% to 90.1%. For the T_1_ states of BF-oTCz and BF-oPCz, significant LE components appear (36.6% to 41.5%), while the LE character in the T_1_ state of BF-oTMCz remains very limited (9.9%), in accordance with its S_1_ counterpart. The lowest-lying ^3^CT state and the lowest-lying ^3^LE state are close in energy for all 3 compounds. Thus, ^3^CT and ^3^LE will inevitably mix with each other, to form T_1_, which are in accordance with the theoretical predictions (vide supra, Fig. S3) [60]. The obviously different orbital nature of T_1_ states originate from the different energy level alignments of the ^3^CT and ^3^LE states, namely, Δ*E*(^3^CT–^3^LE). The lowest ^3^LE state depends on the longest π-conjugation length in the whole molecule. All the 3 molecules adopt very similar configurations and consist of the same π-extended dibenzofuran subunit on which the lowest LE transitions probably occur. Therefore, the lowest ^3^LE states of these 3 molecules should be very close in energy (~2.88 eV, vide infra). However, their lowest CT energies are different due to the different donor strengths. The Δ*E*_ST_ values were theoretically calculated to be 0.14, 0.14, and 0.03 eV for BF-oTCz, BF-oPCz, and BF-oTMCz, respectively. Obviously, the much smaller Δ*E*_ST_ value of BF-oTMCz compared with those of the other 2 compounds can be attributed to the much less ^3^LE contribution in the T_1_ state.

### Photophysical properties

The UV-Vis absorption spectra were recorded in dilute toluene solutions of these materials (10^−5^ M) (Fig. S4). The strong absorption bands ranging from 290 to 380 nm can be attributed to the n–π* and π–π* transitions localized on dibenzofuran and carbazole derivatives, and the weak absorptions in the wavelength region of 380 to 470 nm are assigned to the D–A intramolecular charge transfer (ICT) transitions from carbazole units to dimesitylboron–dibenzofuran units. The solvent effects on PL behaviors were studied by comparing PL spectra of these emitters in various solvents. As shown in Fig. S4, the PL spectra of all 3 emitters display obvious bathochromic shifts and broadened spectrum profiles by increasing the solvent polarity, indicating the CT characteristics of their emissive states. The compound BF-oTMCz shows the most significant solvent effect because of its more CT components.

**Fig. 2.**
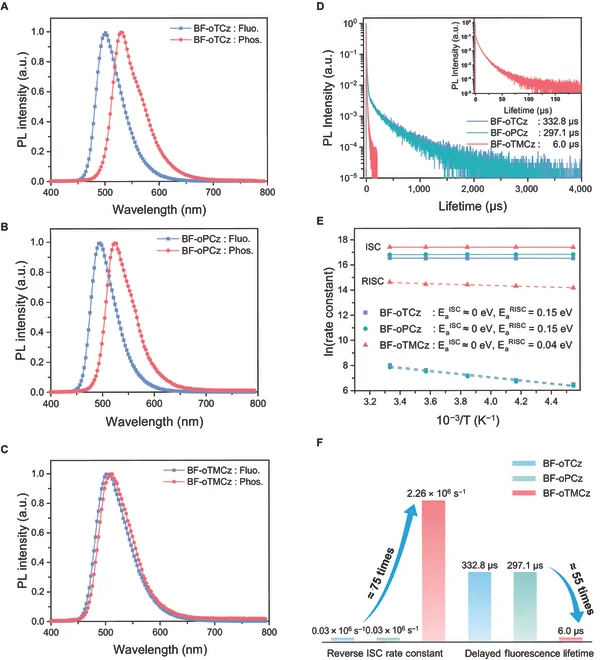
(A to C) Time-resolved PL spectra of BF-oTCz, BF-oPCz, and BF-oTMCz in 20 wt%-doped BCPO films at 77 K. Fluo.: fluorescence; Phos.: phosphorescence. (D) Transient PL decay curves of BF-oTCz, BF-oPCz, and BF-oTMCz in 20 wt%-doped BCPO films. (E) Arrhenius plots of ISC (solid line) and RISC (dashed line), the activation energies of ISC and RISC. (F) Comparison of RISC rate constants (k_RISC_) and delayed fluorescence lifetime (τ_DF_) of BF-oTCz, BF-oPCz, and BF-oTMCz.

To gain insight into the excited state nature, the time-resolved PL (fluorescence and phosphorescence) spectra of these emitters were recorded in 20 wt%-doped BCPO films (BCPO = bis-4-(N-carbazolyl)phenyl)phenylphosphine oxide) at 77 K (Fig. 2A to C) [61]. The fluorescence spectra of all 3 compounds are broad and structureless, confirming that the S_1_ states possess predominant CT nature in approximate energy levels, which are consistent with the solvatochromism phenomena of these compounds (Fig. S4).

The phosphorescence spectra of BF-oTCz and BF-oPCz are strongly redshifted relative to the fluorescence spectra and are both asymmetrical, each showing a shoulder band in addition to the main CT band, which suggests that the T_1_ states of these 2 emitters are hybrid local-charge transfer excited state (^3^HLCT). In contrast, the T_1_ state of BF-oTMCz should be a typical CT state according to its nearly symmetric and slightly broader phosphorescence spectrum. These assignments are in line with theoretical predictions (vide supra, Fig. S3). From these time-resolved PL spectra (Fig. 2A to C), S_1_ and T_1_ energies and Δ*E*_ST_ are estimated to be 2.66, 2.51, and 0.15 eV for BF-oTCz, 2.68, 2.53, and 0.15 eV for BF-oPCz, and 2.65, 2.61, and 0.04 eV for BF-oTMCz, respectively. The photophysical properties of these emitters were further investigated in the 20 wt%-doped BCPO films. The film samples of BF-oTCz, BF-oPCz, and BF-oTMCz exhibit bluish-green to green emission with CT-type steady-state PL spectra peaking at 508, 502, and 515 nm, respectively (Fig. S5a). The transient PL decay characteristics of these doped films at 300 K (Fig. 2D) reveal that each compound shows a typical 2-component TADF decay behavior consisting of a prompt fluorescence (PF) component and a delayed fluorescence (DF) component. The lifetimes of the PF (τ_PF_) and the DF (τ_DF_) are 24.2 ns and 332.8 μs for BF-oTCz, 20.4 ns and 297.1 μs for BF-oPCz, and 25.6 ns and 6.0 μs for BF-oTMCz, respectively. Impressively, the DF lifetime of BF-oTMCz is only ~1/55 and ~1/50 of those of BF-oTCz and BF-oPCz, respectively. Moreover, BF-oTMCz has a much stronger DF contribution to the entire emission, as compared with the other 2 materials.

As listed in Table 1, the overall PLQYs (Φ_PL_) of BF-oTCz, BF-oPCz, and BF-oTMCz in doped BCPO films reach 96.3%, 89.5%, and 94.8% at room temperature, which include quantum efficiencies of 79.7%, 74.8%, and 7.1% for PF (Φ_PF_) and 16.6%, 14.7%, and 87.7% for DF (Φ_DF_), respectively. The much higher TADF contribution and much shorter TADF lifetime generally suggest a much more efficient RISC process for BF-oTMCz. Based on the PLQYs and lifetimes at 300 K, the ISC rate constants (*k*_ISC_) and the RISC rate constants (*k*_RISC_) are estimated to be approximately 8.37 × 10^6^ s^−1^ and 0.03 × 10^6^ s^−1^ for BF-oTCz, 1.24 × 10^7^ s^−1^ and 0.03 × 10^6^ s^−1^ for BF-oPCz, and 3.63 × 10^7^ s^−1^ and 2.26 × 10^6^ s^−1^ for BF-oTMCz, respectively, using a previously reported method (Table 1, see the Supplementary Materials for details) [62]. Notably, although having similar molecular structures, the *k*_RISC_ of BF-oTMCz is approximately 75 times higher than those of BF-oTCz and BF-oPCz. Chou and coworkers have recently reported that the host–guest interaction plays a critical role in boosting TADF emission of multiple-resonance TADF emitters [63]. They have demonstrated that the host–guest interaction that boosts TADF depends on the alignment of orbital energy between the host and guest. Here, in order to investigate the effect of host–guest interaction on the RISC, we selected bis[2-(diphenylphosphino)phenyl]ether oxide (DPEPO) as a control host to prepare doped films of the TADF emitters. Compared with the host BCPO (T_1_ = 3.0 eV), DPEPO has a rather high triplet state energy (3.3 eV), which would prevent significant host–guest interaction [63]. The photophysical properties of the doped DPEPO films were measured and summarized in Fig. S8 and Table S6. The *k*_RISC_ of BF-oTCz, BF-oPCz, and BF-oTMCz in 20 wt%-doped DPEPO films was calculated to be 0.06 × 10^6^, 0.05 × 10^6^, and 2.27 × 10^6^ s^−1^, respectively, which are comparable to those of the 20 wt%-doped BCPO films (0.03 × 10^6^, 0.03 × 10^6^, and 2.26 × 10^6^ s^−1^). These results suggest that the spin-flipping RISC of these TADF emitters is not significantly dependent on host–guest interactions.

**Table 1.** Photophysical data in 20 wt%-doped BCPO films (excited at 355 nm) and calculated reorganization energies of the investigated emitters.

Emitter	λ_PL_^a^	Φ_PL_/Φ_PF_/Φ_DF_^b^	*τ*_PF_/*τ*_DF_^c^	*k*_ISC_/*k*_RISC_^d^	*E*_S_/*E*_T_/Δ*E*_ST_^e^	λ_ISC_/λ_RISC_^f^
	nm	%	ns/μs	10^6^ s^−1^	eV	eV
BF-oTCz	508	96.3/79.7/16.6	24.2/332.8	8.37/0.03	2.66/2.51/0.15	0.172/0.210
BF-oPCz	502	89.5/74.8/14.7	20.4/297.1	12.4/0.03	2.68/2.53/0.15	0.277/0.310
BF-oTMCz	515	94.8/7.10/87.7	25.6/6.0	36.3/2.26	2.65/2.61/0.04	0.005/0.004

^a^ The wavelength at PL maximum. ^b^ Φ_PL_, Φ_PF_, and Φ_DF_ are the overall PLQY, the quantum yields of prompt fluorescence, and delayed fluorescence, respectively. ^c^ τ_PF_ and τ_DF_ are the lifetimes of prompt fluorescence and delayed fluorescence, respectively. ^d^ *k*_ISC_ and *k*_RISC_ represent the rate constants of intersystem crossing (ISC) and reverse ISC (RISC). ^e^ Energy levels of S_1_ and T_1_ were estimated from fluorescence and phosphorescence spectra at 77 K; energy gaps between lowest singlet and triplet excited states (Δ*E*_ST_) were obtained from *E*_S_ − *E*_T_. ^f^ Reorganization energies of ISC and RISC obtained from theoretical calculations.

### Decisive factors for the RISC process

**Fig. 3.**
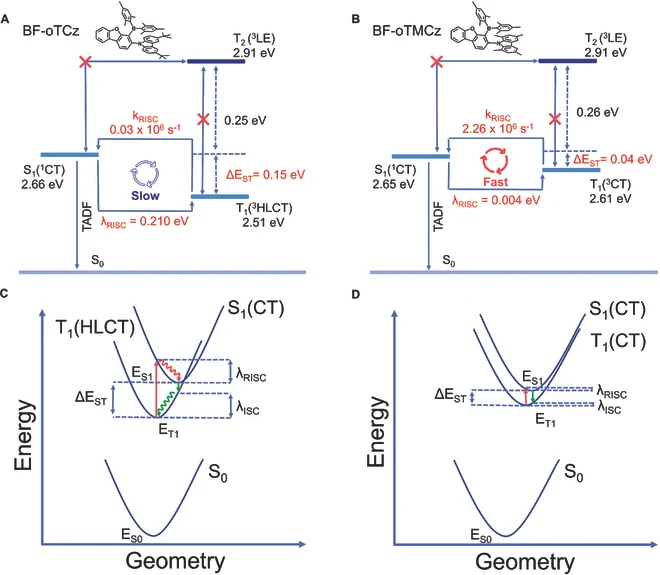
Schematic illustration of TADF mechanism for (A) BF-oTCz and (B) BF-oTMCz. (C and D) Schematic illustration of ISC and RISC processes for (C) BF-oTCz and (D) BF-oTMCz. λ_ISC_ and λ_RISC_ represent the reorganization energies of ISC and RISC, respectively.

Most previous studies attributed the fast RISC process to small Δ*E*_ST_, and especially to the enhanced SOC between the lowest ^1^CT states and the close-lying ^3^LE states. As mentioned above, the TD-DFT calculations revealed that the lowest ^3^LE states mainly reside on the BF acceptor moieties in our TADF molecules. To further confirm the lowest ^3^LE energies of these TADF molecules, the phosphorescence spectra of all the donor units (TCz for 3,6-Di-tert-butyl-carbazole, PCz for 3,6-Biphenyl-carbazole, and TMCz for 1,3,6,8-Tetramethyl-carbazole) and the BF acceptor unit were recorded (Fig. S6). Based on these phosphorescence spectra, the T_1_ energy levels of TCz, PCz, TMCz, and BF were estimated to be 3.06, 2.91, 3.03, and 2.91 eV, respectively. The acceptor moiety (BF) exhibits the lowest T_1_ energy among all the donor and acceptor moieties used for constructing these TADF molecules, suggesting that the lowest ^3^LE of all these TADF molecules probably reside on the BF unit. Accordingly, based on the combination of these theoretical and experimental results, the T_2_ (^3^LE) energy levels of the TADF emitters could be estimated from the phosphorescence spectrum of BF with a value of 2.91 eV, which agree well with the theoretically predicted T_2_ energy levels (2.85 to 2.88 eV, Table S2). In order to figure out the origin of the distinct RISC processes of these materials, we investigated the temperature dependence of *k*_ISC_ and *k*_RISC_. As depicted in Fig. 2e, the *k*_RISC_ values of all the 3 TADF materials decreased with temperature. Based on the Arrhenius equation $\left[k_{\mathrm{RISC}}=A\cdot \text{exp}\left(-\frac{E_{a}}{k_{B}T}\right)\right]$, where *A* is the prefactor, *k*_B_ is the Boltzmann constant, and *T* is temperature, the activation energies of RISC (*E*_a_^RISC^) for BF-oTCz, BF-oPCz, and BF-oTMCz were estimated to be 0.15, 0.15, and 0.04 eV, respectively, which are equal to the corresponding Δ*E*_ST_ values determined from the fluorescence and phosphorescence spectra. In contrast to *k*_RISC_, the *k*_ISC_ of these materials does not show any temperature dependence (Fig. 2E); that is, the activation energies (*E*_a_^ISC^) are zero. The combination of these experimental results suggests that, for all these materials, the upper-lying states, e.g., T_2_ (^3^LE), are not directly involved in both the ISC and RISC processes between S_1_ and T_1_ (Fig. 3A and B). In addition, the energy of Δ*E*_T1_-_T2_ is large (around 0.3 to 0.4 eV); thus, the reverse IC between T_1_ and T_2_ is assumed to be very slow, inhibiting the T_1_–T_2_ (^3^LE)–S_1_ (^1^CT) process. In this case, it seems uncommon that the RISC process (between ^3^CT and ^1^CT states with the same transition character) of BF-oTMCz is much higher than those (RISC between^3^HLCT and ^1^CT) of BF-oTCz and BF-oPCz, because the ^3^CT–^1^CT transition is usually neglected according to the El-Sayed rules. We could rationalize this observation with the Δ*E*_ST_, the RISC reorganization energies, and the non-zero ^3^CT–^1^CT SOC in inhomogeneous media, based on the Marcus–Levich equation (Eq. 1). First, the much smaller Δ*E*_ST_ of BF-oTMCz compared with those of BF-oTCz and BF-oPCz contributes to its high RISC rate. Second, the reorganization energies of RISC and ISC processes of these materials were theoretically calculated. As shown in Table 1, both the RISC reorganization energy (λ_3CT–1CT_ = 0.004 eV) and the ISC reorganization energy (λ_1CT–3CT_ = 0.005 eV) of BF-oTMCz are much lower than those of BF-oTCz (λ_3HLCT–1CT_ = 0.210 eV, λ_1CT–3HLCT_ = 0.172 eV) and BF-oPCz (λ_3HLCT–1CT_ = 0.310 eV, λ_1CT–3HLCT_ = 0.277 eV). According to Eq. 1, a small RISC reorganization energy is conducive to realizing fast RISC. Third, as demonstrated by Park et al. [42], the statistically weighted SOC between the ^3^CT and ^1^CT states that have the same transition character is non-negligible by taking into account the contribution of rotamers, especially in inhomogeneous media [42].

Based on the Marcus–Levich equation (Eq. 1) and the theoretically obtained values of Δ*E*_ST_, RISC reorganization energy and SOC, the *k*_RISC_ values of BF-oTCz, BF-oPCz, and BF-oTMCz were calculated to be 7.30 × 10^4^, 4.32 × 10^4^, and 1.43 × 10^6^ s^−1^, respectively (Table S3). These values are in good agreement with the experimentally obtained *k*_RISC_ values (Table 1). In order to distinguish the contributions of Δ*E*_ST_, RISC reorganization energy, and SOC to *k*_RISC_, we summarized the theoretically obtained values of $\left|<S_{1}\right|{\hat{H}}_{\mathrm{SOC}}{\left|T_{1}>\right|}^{2}$, $\sqrt{\frac{\pi }{\lambda_{\mathrm{RISC}}k_{B}T}}\exp\left[-\frac{{\left(\Delta E_{\mathrm{ST}}+\lambda_{\mathrm{RISC}}\right)}^{2}}{4\lambda_{\mathrm{RISC}}k_{B}T}\right]$ (herein, the contributions of Δ*E*_ST_ and λ_RISC_ are considered as a whole), and *k*_RISC_ in Table S3. It can be found that the $\left|<S_{1}\right|{\hat{H}}_{\mathrm{SOC}}{\left|T_{1}>\right|}^{2}$ value of BF-oTMCz is about one order of magnitude smaller, while the $\sqrt{\frac{\pi }{\lambda_{\mathrm{RISC}}k_{B}T}}\exp\left[-\frac{{\left(\Delta E_{\mathrm{ST}}+\lambda_{\mathrm{RISC}}\right)}^{2}}{4\lambda_{\mathrm{RISC}}k_{B}T}\right]\;$value of BF-oTMCz is about 125 to 296 times larger than those of the other 2 TADF molecules. Therefore, we can conclude that the much higher *k*_RISC_ observed for BF-oTMCz as compared to BF-oTCz and BF-oPCz can be primarily attributed to the contributions of Δ*E*_ST_ and RISC reorganization energy, rather than the SOC between S_1_ and T_1_.

### OLED performance

**Fig. 4.**
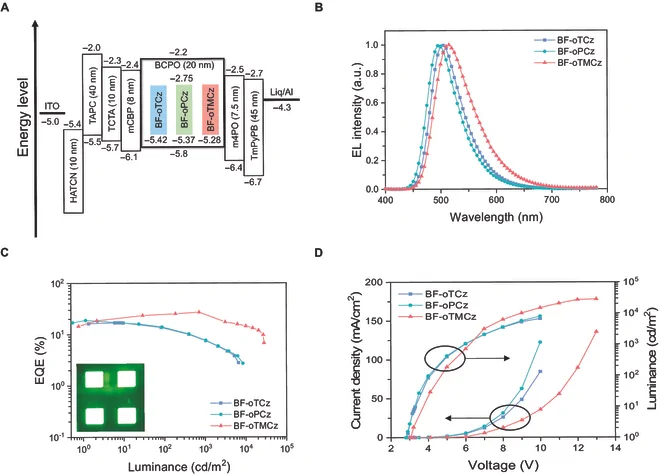
(A) Energy-level diagram of the OLEDs. (B) EL spectra taken at 6 V. (C) External quantum efficiency (EQE) versus luminance characteristics. (D) Current density (*J*)–voltage (*V*)–luminance (*L*) characteristics.

To investigate the electroluminescence (EL) performances of these TADF compounds, we fabricated and tested OLEDs using BF-oTCz, BF-oPCz, and BF-oTMCz as the emitters. Figure 4A depicts the optimized device configuration: ITO/HATCN (10 nm)/TAPC (30 nm)/TCTA (10 nm)/mCBP (8 nm)/BCPO: 20 wt% emitters (20 nm)/m4PO (7.5 nm)/TmPyPB (45 nm)/Liq (2 nm)/Al (100 nm). The related molecular structures are shown in Fig. S12. The graphic and numerical data of device performances are shown in Fig. 4B to D, Fig. S13, and Table 2, respectively. The OLEDs employing all these TADF emitters turned on (luminance = 1 cd/m^2^) at a low voltage of around 2.9 V, indicating the effective charge injection and transport with the well-matched HOMO levels and LUMO levels of the functional layers. These devices showed strong bluish-green to green EL with excellent color stability over a wide range of operating voltages from 3 to 10 V (Fig. S14). The unstructured EL spectra of BF-oTCz-, BF-oPCz-, and BF-oTMCz-based OLEDs peaked at 505, 499, and 515 nm, respectively, which are in accordance with the PL counterparts. No additional emission from the host material or other functional layers indicates that the electrogenerated excitons can be well confined on the dopants.

**Table 2.** Summary of device performances.

Emitter	λ_EL_^a^	*V*_on_^b^	*L*_max_^c^	EQE_max_/EQE_1000_^d^	CE_max_^e^	PE_max_^f^	CIE^g^
	nm	V	cd/m^2^	%	cd/A	lm/W	(*x*, *y*)
BF-oTCz	505	2.9	6,616	17.0/7.7	50.6	49.7	(0.217, 0.543)
BF-oPCz	499	2.9	8,478	18.9/7.8	51.6	56.0	(0.194, 0.482)
BF-oTMCz	515	2.9	28,150	27.1/26.0	85.2	58.3	(0.285, 0.572)

^a^ Turn-on voltage at 1 cd/m^2^. ^b^ Wavelength at EL maximum (at 6 V). ^c^ Maximum luminance (*L*_max_). ^d^ Maximum external quantum efficiencies (EQE_max_); EQE at 1,000 cd/m^2^. ^e^ Maximum current efficiency (CE_max_). ^f^ Maximum power efficiency (PE_max_). ^g^ CIE coordinates measured at 6 V.

The OLED based on BF-oTMCz achieved a very high maximum external quantum efficiency (EQE_max_) of 27.1% and a maximum luminance (*L*_max_) of 28,150 cd/m^2^. In comparison, much smaller EQE_max_ values of 17.0% and 18.9% and much lower *L*_max_ values of 6,616 cd/m^2^ and 8,478 cd/m^2^ were obtained for the BF-oTCz- and BF-oPCz-based OLEDs, respectively. Considering the approximately equal PLQYs of the dopants and the same device configuration, the obvious difference in the above device performances should be mainly attributed to the notably different RISC rates and exciton lifetimes of TADF emitters. The long TADF lifetimes of BF-oTCz (332.8 μs) and BF-oPCz (297.1 μs) would lead to severe exciton annihilation in the emitting layer and thus reduce efficiency and luminance. In particular, the exciton annihilation process would be dramatically exacerbated at high excitation densities. For instance, at a high luminance of 1,000 cd m^−2^, the BF-oTMCz-based OLED maintains an EQE value of 26.0% with an ideal efficiency roll-off of 4.1%. In contrast, the EQEs of the BF-oTCz- and BF-oPCz-based devices dramatically decreased by nearly 60% to 7.7% and 7.8% at 1,000 cd/m^2^, respectively. The sharp contrast between these OLEDs reveals that the RISC rates and exciton lifetimes of the TADF dopants play a critical role in determining device performance.

The BF-oTMCz-based OLED achieved a very high maximum external quantum efficiency (EQE_max_) of 27.1% and a maximum luminance (*L*_max_) of 28,150 cd/m^2^. In comparison, much smaller EQE_max_ values of 17.0% and 18.9% and much lower *L*_max_ values of 6,616 cd/m^2^ and 8,478 cd/m^2^ were obtained for the BF-oTCz- and BF-oPCz-based OLEDs, respectively. Considering the approximately equal PLQYs of the dopants and the same device configuration, the obvious difference in the above device performances should be mainly attributed to the markedly different RISC rates and exciton lifetimes of TADF emitters, which are attributed to the fast spin-flip ^3^CT–^1^CT transition. The long TADF lifetimes of BF-oTCz (332.8 μs) and BF-oPCz (297.1 μs) would lead to severe exciton annihilation in the emitting layer and thus reduce efficiency and luminance. In particular, the exciton annihilation process would be dramatically exacerbated at high excitation densities. For instance, at a high luminance of 1,000 cd m^−2^, the BF-oTMCz-based OLED maintains an EQE value of 26.0% with an ideal efficiency roll-off of 4.1%. In contrast, the EQEs of the BF-oTCz- and BF-oPCz-based devices dramatically decreased by nearly 60% to 7.7% and 7.8% at 1,000 cd/m^2^, respectively. The sharp contrast between these OLEDs reveals that the RISC rates and exciton lifetimes of the TADF dopants, which originate from the fast spin-flipping between ^3^CT and ^1^CT without involvement of the upper-lying LE states, play a critical role in determining device performance.

## Discussion

In summary, we designed and synthesized 3 new TADF materials, namely, BF-oTCz, BF-oPCz, and BF-oTMCz, consisting of the same boron acceptor and different carbazole-derived donors. A detailed photophysical study of these TADF emitters reveals insight into their emission behaviors. Impressively, all these emitters possess similar molecular structures, similar high PLQYs (89.5% to 96.3%), and approximate energy levels of S_1_, but significantly different spin-flipping RISC rates and exciton lifetimes. For BF-oTCz and BF-oPCz that have partial ^3^LE contribution in the T_1_ states, the spin-flipping RISCs are very slow because of the relatively large energy gaps and high reorganization energies between the ^3^HLCT and ^1^CT states, even though the SOC interactions between the states of different transitions are generally considered to be effective. In contrast, BF-oTMCz with more CT components exhibits a much faster RISC transition and a much shorter exciton lifetime as compared with the other 2 emitters, via the fast spin-flipping ^3^CT–^1^CT transition by strikingly reduced Δ*E*_ST_ and reorganization energy, not needing the nonessential participation of an intermediate ^3^LE state. Experimental and theoretical evidences revealed that the small energy gap and low reorganization energy between the ^3^CT and ^1^CT states could provide very fast RISC, without the participation of the upper-lying ^3^LE states. As a result, the OLED based on BF-oTMCz achieved excellent performances with a high EQE over 27.1%, a very small efficiency roll-off of 4.1% at 1,000 cd/m^2^, and a considerably high luminance of 28,150 cd/m^2^, which are markedly superior to the devices employing the other 2 emitters.

Our study reveals that the partial participation of ^3^LE states in the low-lying states could be adverse to attaining fast RISC, owing to the enlarged Δ*E*_ST_ and reorganization energy, and the feasibility of fast spin-flip between ^3^CT–^1^CT transition. To design high-performance TADF-OLED emitters, it is an effective strategy and deserves more attention to accelerate RISC process through the fast spin-flipping ^3^CT–^1^CT transition by reducing the Δ*E*_ST_ and the reorganization energy, without the nonessential participation of an intermediate ^3^LE state. These results provide a new understanding and a direction toward exploring TADF materials with fast RISC process, which are highly desirable for the construction of stable and efficient OLEDs.

## Materials and Methods

Detailed material synthesis and characterization, x-ray structure, computational methodology and results, photophysical properties, analysis of rate constants, TGA and DSC curves, cyclic voltammetry, structure, and performance of devices are included in the Supplementary Materials.

## Data Availability

All data supporting the findings of this study are presented in the article and supplementary materials. Additional data are available from the corresponding author upon reasonable request.

## Supplementary Materials

Supplementary 1 Experiments including general materials and instrumentations. Methods of single crystal analysis, theoretical simulations, device fabrication, and measurement. Syntheses and characterizations of new compounds. Scheme S1. Synthetic procedure. Table S1. Crystal data. Table S2. Calculated energy levels of the excited states, spin-orbit coupling (SOC) constants, and reorganization energies of ISC and RISC. Table S3. Calculated relative data of reverse intersystem crossing rates. Tables S4 to S6. Photophysical properties. Table S7. Summary of CV data and energy levels. Table S8. OLED data. Fig. S1. Single-crystal packing diagram. Fig. S2. RDG scatter diagrams. Fig. S3. Electron-density distribution of the lowest excited states. Figs. S4 to S9. Photophysical properties. Fig. S10. Thermal properties. Fig. S11. Cyclic voltammograms. Figs. S12 to S16. OLED data. Figs. S17 to S26. NMR spectra.
