# Supplementary material for: Efficient Spin-Flip between Charge-Transfer States for High-Performance Electroluminescence, without an Intermediate Locally Excited State
Source: Research (Wash D C). 2023 May 26;6:0155. doi: 10.34133/research.0155 (PMC10214979; doi:10.34133/research.0155)
Supplement: Supplementary 1 — Experiments including general materials and instrumentations. Methods of single crystal analysis, theoretical simulations, device fabrication, and measurement. Syntheses and characterizations of new compounds. Scheme S1. Synthetic procedure. Table S1. Crystal data. Table S2. Calculated energy levels of the excited states, spin-orbit coupling (SOC) constants, and reorganization energies of ISC and RISC. Table S3. Calculated relative data of reverse intersystem crossing rates. Tables S4 to S6. Photophysical properties. Table S7. Summary of CV data and energy levels. Table S8. OLED data. Fig. S1. Single-crystal packing diagram. Fig. S2. RDG scatter diagrams. Fig. S3. Electron-density distribution of the lowest excited states. Figs. S4 to S9. Photophysical properties. Fig. S10. Thermal properties. Fig. S11. Cyclic voltammograms. Figs. S12 to S16. OLED data. Figs. S17 to S26. NMR spectra. [file research.0155.f1.docx]

**Supplementary Materials**

**Efficient Spin-flip between Charge-Transfer States for High-Performance Electroluminescence, Without an Intermediate Locally Excited State**

Donghai Zhang ^1, 2, 3, 4^, Shanshan Jiang ^1, 3^, Xiaodong Tao ^1, 2, 3, 4^, Fulin Lin ^1, 3^, Lingyi Meng ^1, 3^, Xu-Lin Chen ^1, 2, 3^*, Can-Zhong Lu ^1, 2, 3, 4^*

^1^ State Key Laboratory of Structural Chemistry, Fujian Institute of Research on the Structure of Matter, Chinese Academy of Sciences, Fuzhou, Fujian 350002, China.

^2^ Fujian Science & Technology Innovation Laboratory for Optoelectronic Information of China, Fuzhou, Fujian 350108, China.

^3^ Xiamen Key Laboratory of Rare Earth Photoelectric Functional Materials, Xiamen Institute of Rare Earth Materials, Haixi Institutes, Chinese Academy of Sciences, Xiamen, Fujian 361021, China.

^4^ University of Chinese Academy of Sciences, Beijing 100049, China.

* Corresponding authors:

Can-Zhong Lu, E-mail: [*czlu@fjirsm.ac.cn*](mailto:czlu@fjirsm.ac.cn)

Xu-Lin Chen, E-mail: [*xlchem@fjirsm.ac.cn*](mailto:xlchem@fjirsm.ac.cn)

**Table of Contents**

1. General Methods…………………………………………………………………………..……S2

2. Material Synthesis and Characterization…………………………………………..……….…...S2

3. X-ray structure determinations………………………………………………….……..….….....S6

4. Computational methodology and results……………………………………………..............…S8

5. Photophysical Properties……………………………………………….……….……..………S10

6. Thermogravimetric Analysis (TGA) and Differential Scanning Calorimetry (DSC)……….....S14

7. Cyclic Voltammetry………………………….………………………….……….....................S15

8. Device Fabrication and Characterization……………………………………………………....S16

9. NMR Spectra…………………………….……………………….……….……………..….....S20

**1. General Methods**

All air- and moisture-sensitive reactions were carried out under an argon atmosphere. All reagents and solvents were obtained commercially and used without further purification except as noted, purchased from Adamas Reagent, Ltd and Bidepharm, Ltd and Sinopharm Chemical Reagent Co., Ltd . Dry tetrahydrofuran, dry toluene and dichloromethane (DCM) were purchased from Adamas Reagent, Ltd. Toluene was dried over molecular sieves (4 Å) before use. The as-prepared TADF materials were dried by vacuum drying and further purified by sublimation. ^1^H NMR (500 MHz) and ^13^C NMR (126 MHz) spectra were recorded on a Bruker Avance III NMR spectrometer, in deuterated chloroform (CD_1_Cl_3_). Thermogravimetric analyses (TGA) were performed on a METTLER TOLEDO system with a heating rate of 20 °C/min under argon. UV-Vis absorption spectra were recorded with a Agilent Cary 5000 UV-Vis spectrophotometer under ambient condition. The absolute PLQYs were measured on an Edinburgh FLS1000 spectrophotometer equipped with an integrating sphere. Steady-state PL spectra were measured on an Edinburgh FLS980 using a Xenon lamp as an excitation light source. The transient PL decay curves were measured on the same spectrophotometer (FLS980) in multi-channel scaling (MCS) mode with a NT242-1K OPO laser as an excitation light source. The time-resolved PL spectra were recorded on an Edinburgh LP980 spectrophotometer with a NT242-1K OPO laser excitation source.

**2. Material Synthesis and Characterization**


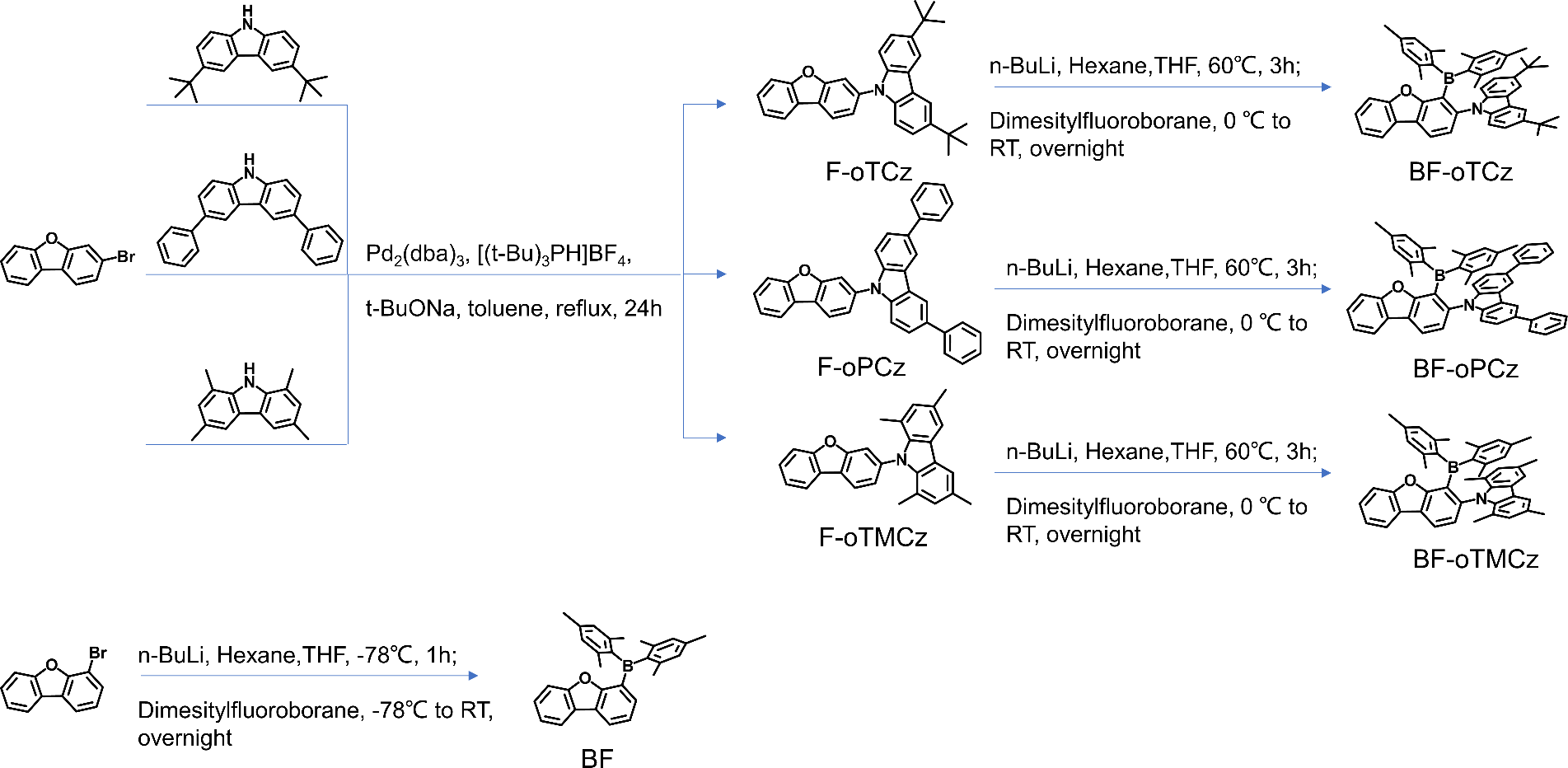


**Scheme S1.** Synthetic route of BF-oTCz, BF-oPCz and BF-oTMCz.

Synthesis of F-oTCz

A mixture of 3-Bromodibenzo[b,d]furan (3.70 g, 15 mmol), 3,6-Di-tert-butylcarbazole (5.59 g, 20 mmol), Pd_2_(dba)_3_ (0.687 g, 0.75 mmol), [(t-Bu)_3_PH]BF_4_ (0.435 g, 1.5 mmol), and t-BuONa (3.60 g, 37.5 mmol) followed by toluene (100 mL) was stirring under argon atmosphere. The reaction solution was heated and refluxed for 24 hours. After the mixture cooled to room temperature, water was added to the reaction mixture at room temperature. The combined organic layer was extracted with DCM and then dried over anhydrous MgSO_4_. After filtration and evaporation, the crude product was purified by column chromatography using Petroleum ether. F-oTCz (4.68 g, 10.5 mmol, 70%) was obtained as white solid. ^1^H NMR (500 MHz, Chloroform-*d*) δ 8.22 (dq, *J* = 6.4, 2.1 Hz, 2H), 8.15 (d, *J* = 8.1 Hz, 1H), 8.06 (d, *J* = 7.7 Hz, 1H), 7.82 (dq, *J* = 4.1, 1.9 Hz, 1H), 7.67 (dd, *J* = 8.2, 2.8 Hz, 1H), 7.60 (ddt, *J* = 6.8, 3.2, 1.7 Hz, 1H), 7.58 – 7.42 (m, 6H), 1.53 (dd, *J* = 5.9, 2.7 Hz, 18H).

Synthesis of F-oPCz

A mixture of 3-Bromodibenzo[b,d]furan (3.70 g, 15 mmol), 3,6-Biphenyl-9H-carbazole (6.39 g, 20 mmol), Pd2(dba)3 (0.687 g, 75 mmol), [(t-Bu)3PH]BF4 (0.435 g, 1.5 mmol), and t-BuONa (3.60 g, 37.5 mmol) followed by toluene (100 mL) was stirring under argon atmosphere. The reaction solution was heated and refluxed for 24 hours. After the mixture cooled to room temperature, water was added to the reaction mixture at room temperature. The combined organic layer was extracted with DCM and then dried over anhydrous MgSO4. After filtration and evaporation, the crude product was purified by column chromatography using Petroleum ether. F-oPCz (5.83 g, 12 mmol, 80 %) was obtained as white solid. ^1^H NMR (500 MHz, Chloroform-*d*) δ 8.47 (t, *J* = 1.9 Hz, 2H), 8.19 (dd, *J* = 8.1, 3.1 Hz, 1H), 8.08 (d, *J* = 7.6 Hz, 1H), 7.86 (t, *J* = 1.6 Hz, 1H), 7.79 (dt, *J* = 8.1, 1.5 Hz, 4H), 7.73 (dd, *J* = 8.5, 1.8 Hz, 2H), 7.69 (d, *J* = 8.1 Hz, 1H), 7.63 (dt, *J* = 8.1, 2.0 Hz, 1H), 7.61 – 7.51 (m, 7H), 7.47 (td, *J* = 7.5, 1.0 Hz, 1H), 7.45 – 7.38 (m, 2H).

Synthesis of F-oTMCz

A mixture of 3-Bromodibenzo[b,d]furan (3.70 g, 15 mmol), 1,3,6,8-tetramethyl-9H-carbazole (3.57 g,16 mmol), Pd2(dba)3 (0.687 g, 75 mmol), [(t-Bu)_3_PH]BF_4_ (0.435 g, 1.5 mmol), and t-BuONa (3.60 g, 37.5 mmol) followed by toluene (100 mL) was stirring under argon atmosphere. The reaction solution was heated and refluxed for 24 hours. After the mixture cooled to room temperature, water was added to the reaction mixture at room temperature. The combined organic layer was extracted with DCM and then dried over anhydrous MgSO4. After filtration and evaporation, the crude product was purified by column chromatography using Petroleum ether. F-oTMCz (5.55 g, 14.3 mmol, 95%) was obtained as white solid. ^1^H NMR (500 MHz, Chloroform-*d*) δ 8.10 – 8.05 (m, 1H), 8.02 (d, *J* = 8.1 Hz, 1H), 7.82 (s, 2H), 7.75 (d, *J* = 1.8 Hz, 1H), 7.67 (d, *J* = 8.2 Hz, 1H), 7.60 – 7.41 (m, 3H), 6.96 (s, 2H), 2.53 (s, 6H), 1.90 (s, 6H).

Synthesis of BF

n‐BuLi in hexanes (2.5M, 2.8 mL, 7 mmol) was added dropwise to a solution of compound 4-Bromodibenzo[b,d]furan (1.48 g, 6 mmol) in dry THF (40 mL) at -78 ℃ under argon atmosphere and stirred at -78 °C for an additional 30 min. Then, dimesitylboron fluoride (1.34 g, 5 mmol) was added at -78 °C, then the mixture was slowly warm to room temperature and stirred overnight at room temperature. The reaction mixture was evaporated in vacuo to remove the solvent. The residue was extracted using DCM and brine, the organic layer was dehydrated using anhydrous sodium sulfate. And concentrated under vacuum. The residue was purified by column chromatography using Petroleum ether. BF was obtained as a white solid (1.67 g, 4.0 mmol, 80 %).^1^H NMR (500 MHz, Chloroform-*d*) δ 8.11 (td, *J* = 7.2, 6.3, 2.4 Hz, 1H), 8.02 – 7.95 (m, 1H), 7.52 – 7.45 (m, 1H), 7.45 – 7.39 (m, 1H), 7.36 (dddd, *J* = 9.4, 7.0, 4.7, 2.3 Hz, 3H), 6.92 – 6.80 (m, 4H), 2.37 (dt, *J* = 9.8, 2.5 Hz, 6H), 2.08 (dt, *J* = 13.4, 3.2 Hz, 13H).

Synthesis of BF-oTCz

n‐BuLi in hexanes (2.5 M, 3.6 mL, 9 mmol) was added dropwise to a solution of compound F-oTCz (3.56 g, 8 mmol) in dry THF (40 mL) at 0℃ under argon atmosphere and stirred at 0 °C for an additional 30 min. The reaction solution was heated and refluxed for 6 hours. Then, the mixture cooled to 0°C, dimesitylboron fluoride (2.15 g, 8 mmol) was added, and then the mixture was stirred overnight at room temperature. The reaction mixture was evaporated in vacuo to remove the solvent. The residue was extracted using DCM and brine, the organic layer was dehydrated using anhydrous sodium sulfate. And concentrated under vacuum. The residue was purified by column chromatography using Petroleum ether. BF-oTCz was obtained as a pale yellow solid (3.12 g, 4.5 mmol, 56.3 %). ^1^H NMR (500 MHz, Chloroform-*d*) δ 8.20 (s, 1H), 8.19 (s, 1H), 8.04 – 8.03 (m, 1H), 8.02 (d, *J* = 1.4 Hz, 1H), 7.86 (s, 4H), 7.45 – 7.42 (m, 2H), 7.39 (d, *J* = 1.2 Hz, 1H), 7.39 (s, 1H), 7.38 (d, *J* = 1.2 Hz, 1H), 7.37 (s, 1H), 7.31 (d, *J* = 0.9 Hz, 1H), 7.30 (d, *J* = 0.9 Hz, 1H), 1.45 (s, 36H). ^13^C NMR (126 MHz, Chloroform-*d*) δ 159.64, 156.76, 141.99, 141.80, 141.50, 140.91, 131.21, 127.23, 125.82, 123.80, 123.52, 123.39, 123.05, 122.77, 122.39, 120.59, 115.18, 112.06, 109.91, 77.32, 77.06, 76.81, 34.63, 32.16, 21.27. Anal. Calcd. for C50H52BNO (%): C 86.56, H 7.56, N 2.02; found: C 86.963, H 7.113, N 2.176.

Synthesis of BF-oPCz

n‐BuLi in hexanes (2.5M, 3.6 mL, 9 mmol) was added dropwise to a solution of compound F-oPCz (3.88 g, 8 mmol) in dry THF (40 mL) at 0℃ under argon atmosphere and stirred at 0 °C for an additional 30 min. The reaction solution was heated and refluxed for 6 hours. Then, the mixture cooled to 0°C, dimesitylboron fluoride (2.15 g, 8 mmol) was added, and then the mixture was stirred overnight at room temperature. The reaction mixture was evaporated in vacuo to remove the solvent. The residue was extracted using DCM and brine, the organic layer was dehydrated using anhydrous sodium sulfate. And concentrated under vacuum. The residue was purified by column chromatography using Petroleum ether. BF-oPCz was obtained as a pale yellow solid (2.20 g, 3 mmol, 37.5 %).^1^H NMR (500 MHz, Chloroform-*d*) δ 8.28 (d, *J* = 7.9 Hz, 1H), 8.17 (s, 2H), 7.76 (d, *J* = 1.5 Hz, 2H), 7.74 (t, *J* = 1.2 Hz, 2H), 7.56 – 7.47 (m, 8H), 7.43 (td, *J* = 7.5, 1.1 Hz, 1H), 7.41 – 7.33 (m, 3H), 7.17 (s, 2H), 6.40 (s, 4H), 2.05 (s, 18H). 13C NMR (126 MHz, Chloroform-*d*) δ 159.58, 156.87, 142.94, 142.31, 141.85, 139.94, 132.77, 131.57, 128.79, 128.08, 127.50, 127.30, 126.41, 125.79, 124.60, 123.99, 123.93, 123.82, 123.79, 123.28, 122.91, 120.72, 118.05, 112.16, 110.99, 77.33, 77.08, 76.83, 21.16. Anal. Calcd. for C54H44BNO (%): C 88.39, H 6.04, N 1.91; found: C 88.998, H 6.048, N 2.055.

Synthesis of BF-oTMCz

n‐BuLi in hexanes (2.5M, 3.6 mL, 9 mmol) was added dropwise to a solution of compound F-oTMCz (3.12 g, 8 mmol) in dry THF (40 mL) at 0℃ under argon atmosphere and stirred at 0 °C for an additional 30 min. The reaction solution was heated and refluxed for 6 hours. Then, the mixture cooled to 0°C, dimesitylboron fluoride (2.15 g, 8 mmol) was added, and then the mixture was stirred overnight at room temperature. The reaction mixture was evaporated in vacuo to remove the solvent. The residue was extracted using DCM and brine, the organic layer was dehydrated using anhydrous sodium sulfate. And concentrated under vacuum. The residue was purified by column chromatography using Petroleum ether. BF-oTMCz was obtained as a pale yellow solid (2.23 g, 3.5 mmol, 44 %). ^1^H NMR (500 MHz, Chloroform-*d*) δ 8.19 (d, *J* = 8.0 Hz, 1H), 8.04 (dd, *J* = 7.5, 1.5 Hz, 1H), 7.97 (d, *J* = 8.0 Hz, 1H), 7.42 (td, *J* = 7.7, 1.5 Hz, 1H), 7.37 (td, *J* = 7.4, 1.2 Hz, 1H), 7.32 (s, 2H), 7.22 (dd, *J* = 7.7, 1.2 Hz, 1H), 6.73 (s, 2H), 6.31 (s, 3H), 2.44 (s, 6H), 2.16 (s, 6H), 1.98 (s, 6H), 1.85 (s, 12H). ^13^C NMR (126 MHz, Chloroform-*d*) δ 159.22, 156.67, 143.78, 141.46, 139.78, 138.14, 129.57, 129.24, 128.47, 127.26, 123.65, 123.24, 122.68, 121.99, 121.30, 120.57, 117.33, 111.96, 77.29, 77.04, 76.78, 21.18, 21.13, 20.70. Anal. Calcd. for C46H44BNO (%): C 86.64, H 6.96, N 2.20; found: C 87.164, H 6.874, N 2.325.

**3.** **X-ray structure determination**

The single crystal of BF-oTCz, BF-oPCz and BF-oTMCz was obtained by slow diffusion of ethanol into a dichloromethane solution of this compound. However, we have failed in our deliberate attempts to crystallize BF-oTCz, BF-oPCz and BF-oTMCz. Single-crystal X-ray diffraction data were collected on a Bruker D8 VENTURE diffractometer equipped with Mo Ka (λ = 0.76 Å) radiation under cryogenic conditions. The crystal was kept at 200 K during data collection. Using Olex2^5^, the structure was solved with the ShelXT6 structure solution program using Intrinsic Phasing and refined with the ShelXL6 refinement package using Least Squares minimization. Hydrogen atoms were added in idealized positions. All non-hydrogen atoms were refined with anisotropic displacement parameters. Details of crystal and structure refinement are listed in **Table S1**. CCDC 2203396, 2203399, 2203400 contains the supplementary crystallographic data for BF-oTCz, BF-oPCz and BF-oTMCz. The data can be obtained free of charge from the Cambridge Crystallographic Data Centre via [www.ccdc.cam.ac.uk/data_request/cif](http://www.ccdc.cam.ac.uk/data_request/cif).

**Table S1.** Crystal data and structure refinement for BF-oTCz, BF-oPCz and BF-oTMCz.

| Compound | BF-oTCz | BF-oPCz | BF-oTMCz |
| --- | --- | --- | --- |
| CCDC Number | 2203396 | 2203399 | 2203400 |
| Empirical formula | C_50_H_52_BNO | C_54_H_44_BNO | C46H44BNO |
| Formula weight | 693.73 | 733.71 | 637.63 |
| Temperature/K | 200 | 200 | 200 |
| Crystal system | orthorhombic | monoclinic | orthorhombic |
| Space group | Pccn | P2/c | Pbca |
| a/Å | 29.4374(16) | 16.7057(6) | 9.8004(2) |
| b/Å | 32.1511(16) | 10.8759(3) | 22.5234(4) |
| c/Å | 17.1667(11) | 22.3013(8) | 31.0938(5) |
| α/° | 90 | 90 | 90 |
| β/° | 90 | 93.048(2) | 90 |
| γ/° | 90 | 90 | 90 |
| Volume/Å3 | 16247.3(16) | 4046.2(2) | 6863.6(2) |
| Z | 16 | 4 | 8 |
| ρcalc g/cm3 | 1.134 | 1.204 | 1.234 |
| μ/mm-1 | 0.066 | 0.070 | 0.072 |
| F(000) | 5952.0 | 1552.0 | 2720.0 |
| Radiation | MoKα (λ = 0.71073) | MoKα (λ = 0.71073) | MoKα (λ = 0.71073) |
| 2Θ range for data collection/° | 4.044 to 63.816 | 3.658 to 55.04 | 4.466 to 54.224 |
| Reflections collected | 239093 | 210371 | 55071 |
| Goodness-of-fit on F2 | 1.019 | 1.038 | 1.059 |
| Final R indexes [I>=2σ (I)] | R1 = 0.0688,  wR2 = 0.1510 | R1 = 0.0475,  wR2 = 0.1109 | R1 = 0.0591,  wR2 = 0.1583 |
| Final R indexes [all data] | R1 = 0.1573,  wR2 = 0.1986 | R1 = 0.0698,  wR2 = 0.1264 | R1 = 0.0840,  wR2 = 0.1863 |

**
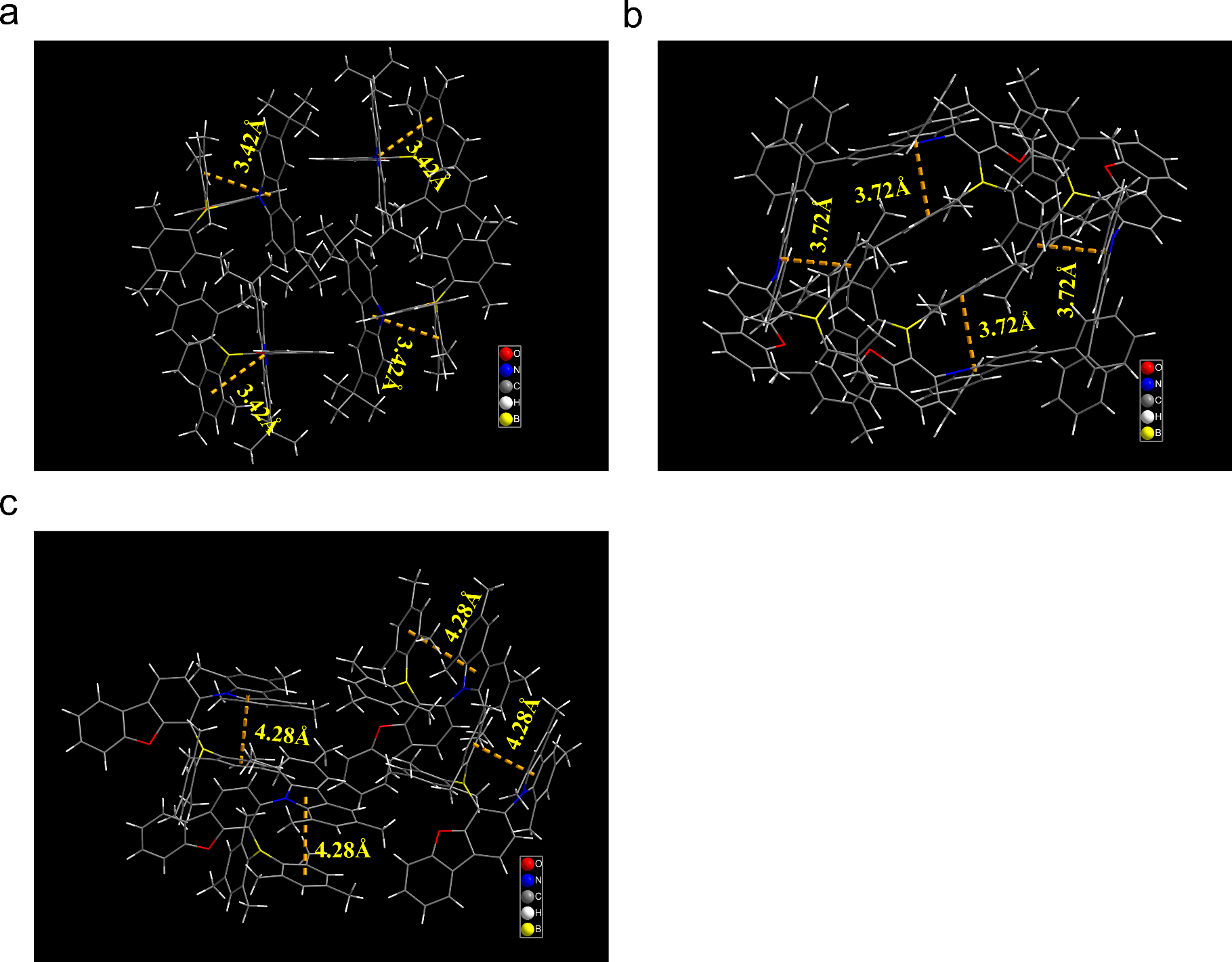
**

**Figure S1**. Single-crystal packing diagram of BF-oTCz(a), BF-oPCz(b) and BF-oTMCz(c).

**4.** **Computational methodology and results**

All the calculations were carried out using the Gaussian 09 program package3. The density functional theory (DFT) calculations at the PBE0/6-311g (d,p) level were used to optimize the ground state geometries of the investigated compounds. Time-dependent density functional theory (TD-DFT) calculations were performed at the same level using the optimized ground state geometries. The spin-orbit couplings were calculated at M06-2X/DKH by ORCA. The electron density diagrams of molecular orbitals were generated using GaussView program. The partition orbital composition was analyzed with the Multiwfn 2.4 program. Reduced density gradient (RDG) is a fundamental dimensionless quantity in DFT used to describe the deviation from a homogeneous electron distribution. The real space function composed of electron density and its derivatives reduced gradient will assume very small values, approaching zero, for regions of both covalent bonding and noncovalent interactions (NCI). Notice that NCI can be isolated as regions with low density and low reduced gradient^3^. Here, RDG function and Sign (λ_2_)ρ, where λ_2_ is the second largest eigenvalue of Hessian matrix of electron density, were calculated and visualized using Multiwfn and VMD. Optimized S_1_ and T_1_ Geometries of BF-oTCz, BF-oPCz and BF-oTMCz using PBE0/6-311g (d,p) for Reorganization Energy Analysis for the conversion between S_1_ and T_1_. We use the molecular materials property prediction package (MOMAP) to calculate the reverse intersystem crossing rates at 300K.

**
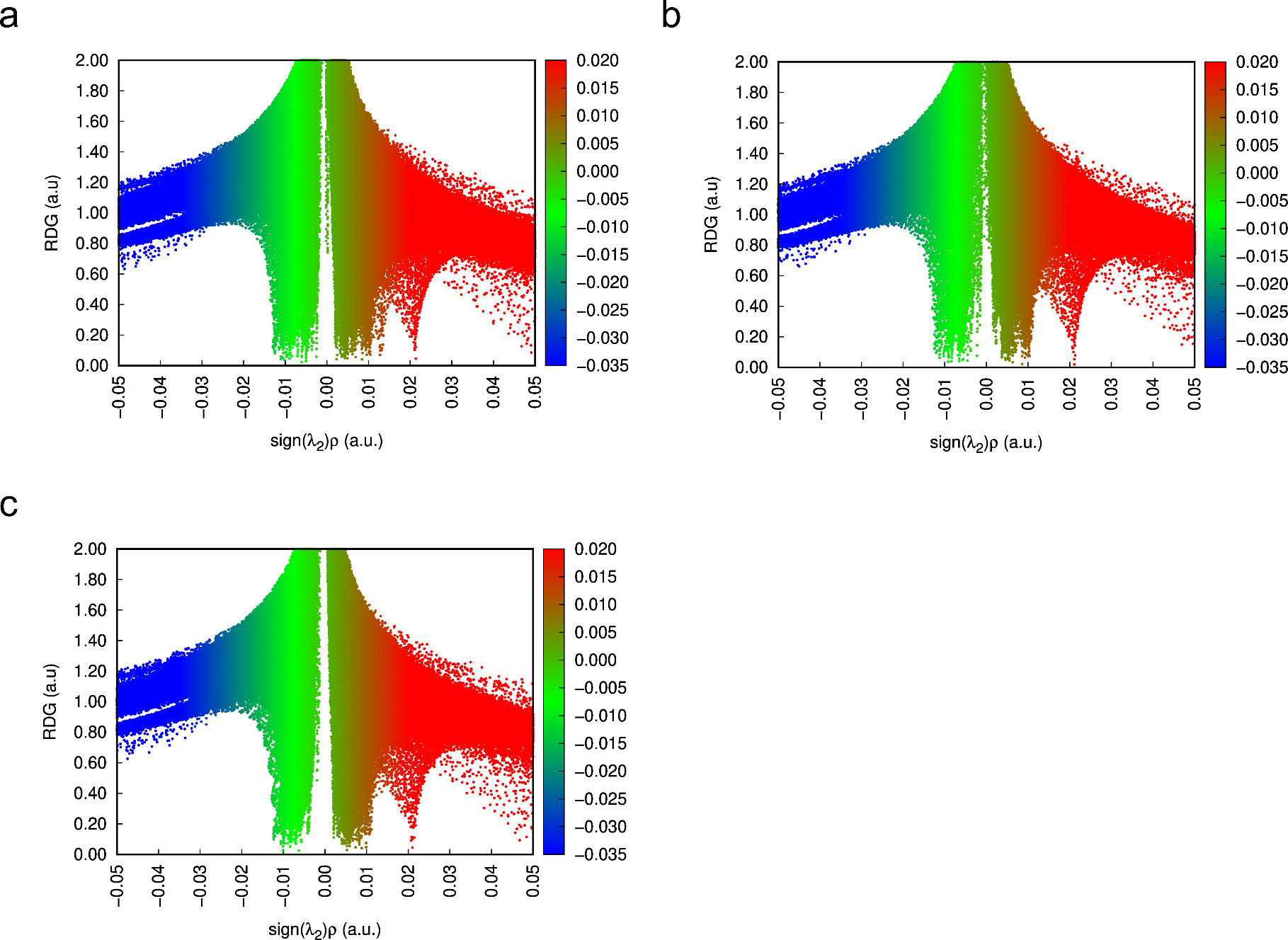
**

**Figure S2**. RDG scatter diagrams BF-oTCz(a), BF-oPCz(b) and BF-oTMCz(c).


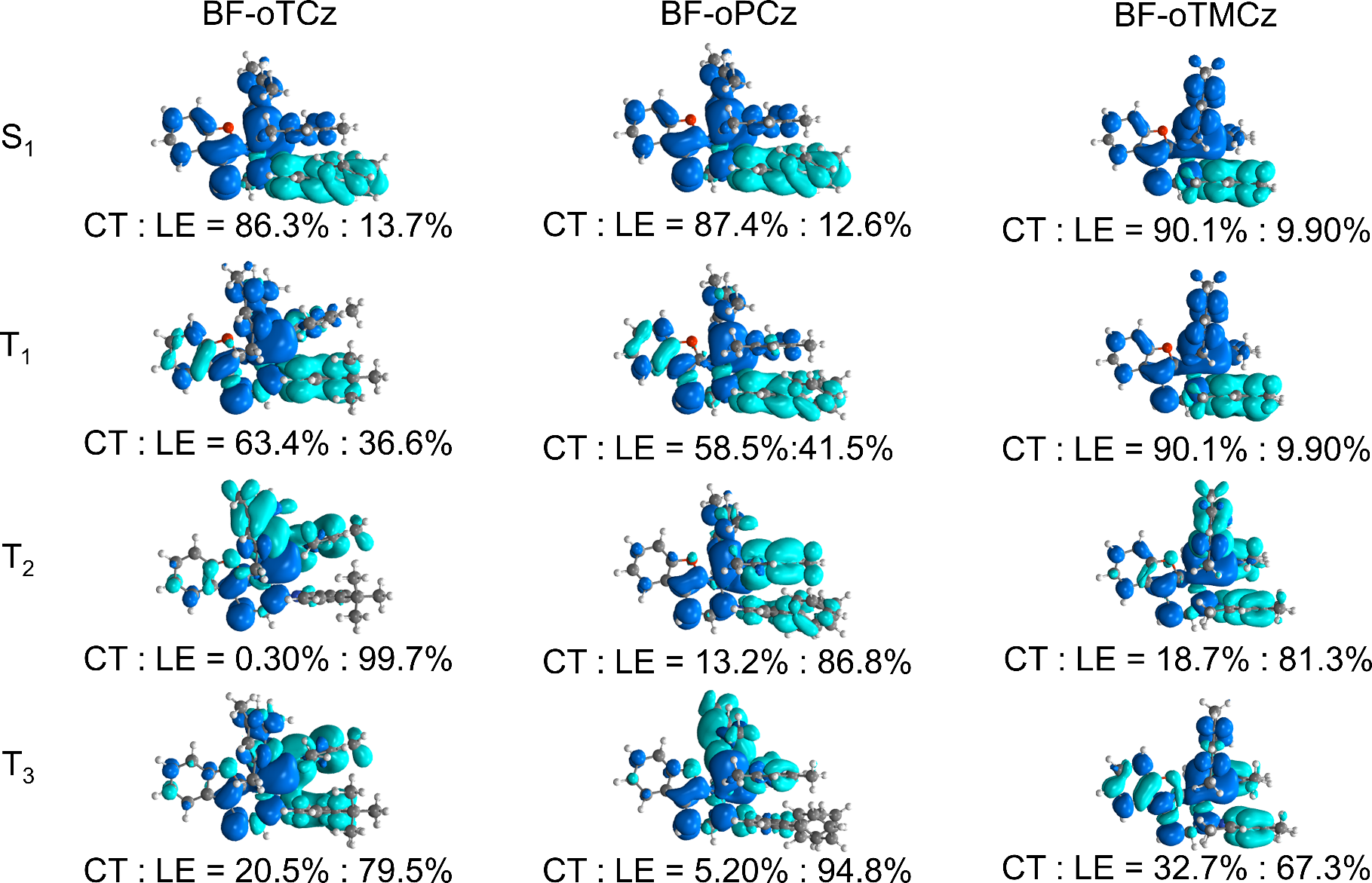


**Figure S3**. Electron-density distribution of the lowest excited states (the green area denotes an increase in charge density, while the blue area denotes a decrease in charge density).

**Table S2.** Calculated energy levels of the excited states, spin-orbit coupling (SOC) constants and reorganization energies of ISC and RISC.

| Compound | S_1_ | T_1_ | T_2_ | T_3_ | SOC_S1-T1_ | SOC_T2-S1_ | SOC_T3-S1_ | λ_ISC_/λ_RISC_ |
| --- | --- | --- | --- | --- | --- | --- | --- | --- |
|  | [eV] | [eV] | [eV] | [eV] | [cm^-1^] | [cm^-1^] | [cm^-1^] | [cm^-1^] |
| BF-oTCz | 2.76 | 2.62 | 2.87 | 2.93 | 0.48 | 0.69 | 0.53 | 0.172/0.210 |
| BF-oPCz | 2.83 | 2.69 | 2.85 | 2.88 | 0.57 | 0.56 | 0.65 | 0.277/0.310 |
| BF-oTMCz | 2.76 | 2.73 | 2.88 | 2.90 | 0.19 | 0.89 | 0.66 | 0.005/0.004 |

**Table S3.** Calculated relative data of spin-orbit coupling (SOC) value, the energy gap between the lowest singlet excited states and the lowest triplet excited states (ΔE_ST_), and reorganization energies of RISC (λ_RISC_) in Marcus-Levich equation (**Equation 1**) at uniform energy units of eV. And the calculated reverse intersystem crossing rates by MOMAP, according to **Equation 1.**

| Compound | SOC_S1-T1_ | $\left\vert<S_{1}\right\vert\hat{H}_{SOC}\left\vert T_{1}>\right\vert^{2}$ | ΔE_ST_/λ_RISC_ | $\sqrt{\frac{\pi}{\lambda_{RISC}k_{B}T}}\exp\left[-\frac{\left(\Delta E_{ST}+\lambda_{RISC}\right)^{2}}{4\lambda_{RISC}k_{B}T}\right]$ | k_RISC_ |
| --- | --- | --- | --- | --- | --- |
|  | [eV] |  | [eV] |  | s^-1^ |
| BF-oTCz | 5.95×10^-5^ | 3.54×10^-9^ | 0.14/0.210 | 0.085 | 7.30×10^4^ |
| BF-oPCz | 7.07×10^-5^ | 5.00×10^-9^ | 0.14/0.310 | 0.036 | 4.32×10^4^ |
| BF-oTMCz | 2.36×10^-5^ | 5.57×10^-10^ | 0.03/0.004 | 10.655 | 1.43×10^6^ |

**5. Photophysical Properties**


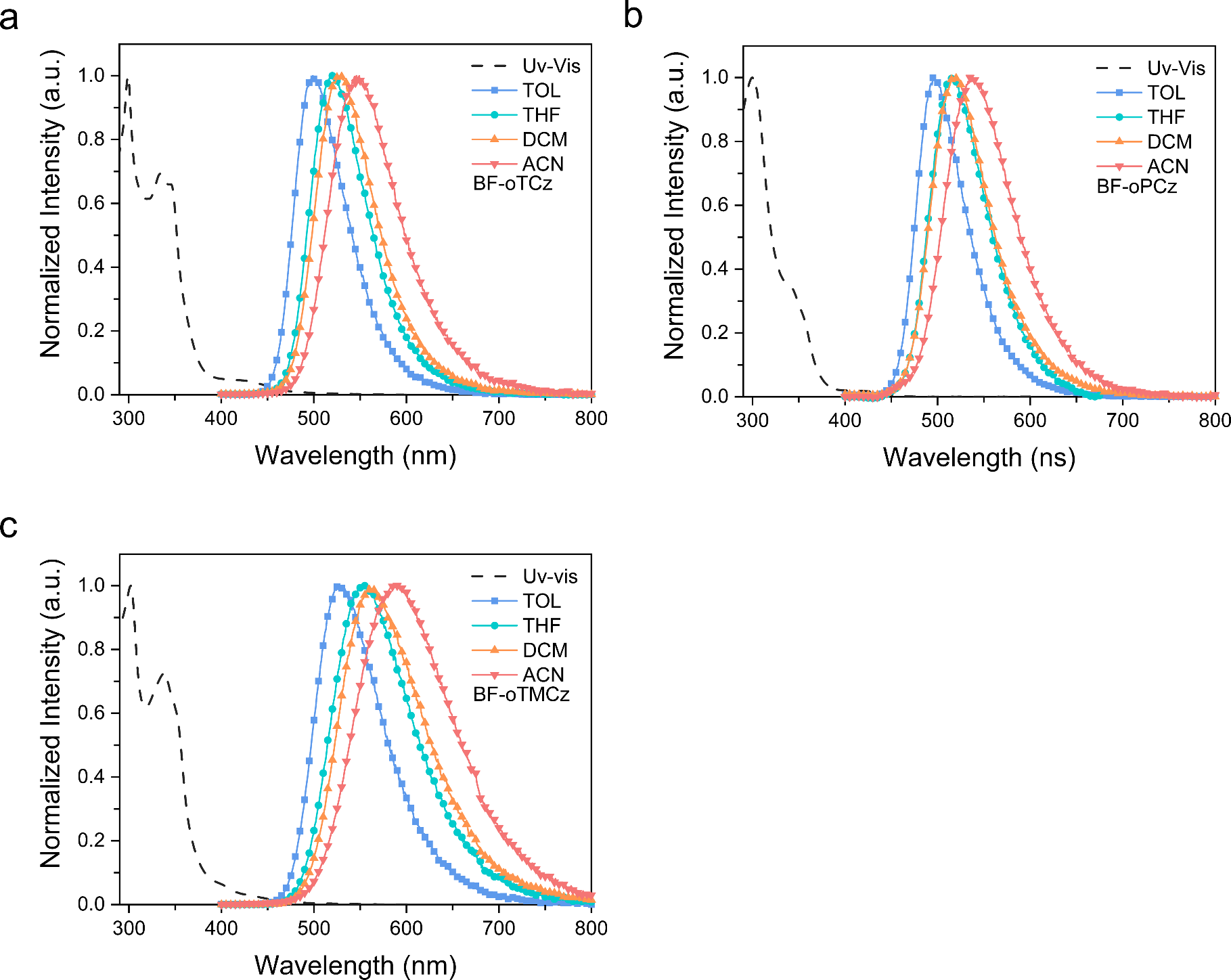


**Figure S4.** Absorption spectra and steady-state PL spectra of BF-oTCz, BF-oPCz and BF-oTMCz, measured at room temperature in various solvents (c = 2.0 × 10^-5^ M).

**Table S4**. UV-vis absorption in toluene; steady-state PL data and stokes shift for BF-oTCz, BF-oPCz and BF-oTMCz in different solvents at room temperature.

| Compound |  | Tol | THF | DCM | ACN |
| --- | --- | --- | --- | --- | --- |
|  | Abs_max_^[a]^ (nm) | 299 | - | - | - |
| BF-oTCz | PL_max_^[b]^ (nm) | 500 | 520 | 527 | 547 |
|  | Stokes shift^[c]^(nm) | 201 | 221 | 228 | 248 |
|  | Abs_max_(nm) | 300 | - | - | - |
| BF-oPCz | PL_max_(nm) | 496 | 517 | 519 | 538 |
|  | Stokes shift(nm) | 196 | 217 | 219 | 238 |
|  | Abs_max_(nm) | 302 | - | - | - |
| BF-oTMCz | PL_max_(nm) | 529 | 555 | 564 | 590 |
|  | Stokes shift(nm) | 227 | 253 | 262 | 288 |

[a] the wavelength at UV-vis absorption maximum in toluene (2 × 10^-5^ M); [b] the wavelength at photoluminescence maximum (excited at 355 nm); [c] Stokes shift is defined as the difference between the maximum absorption wavelength in toluene and maximum emission wavelength in different solvents (in wavelength).


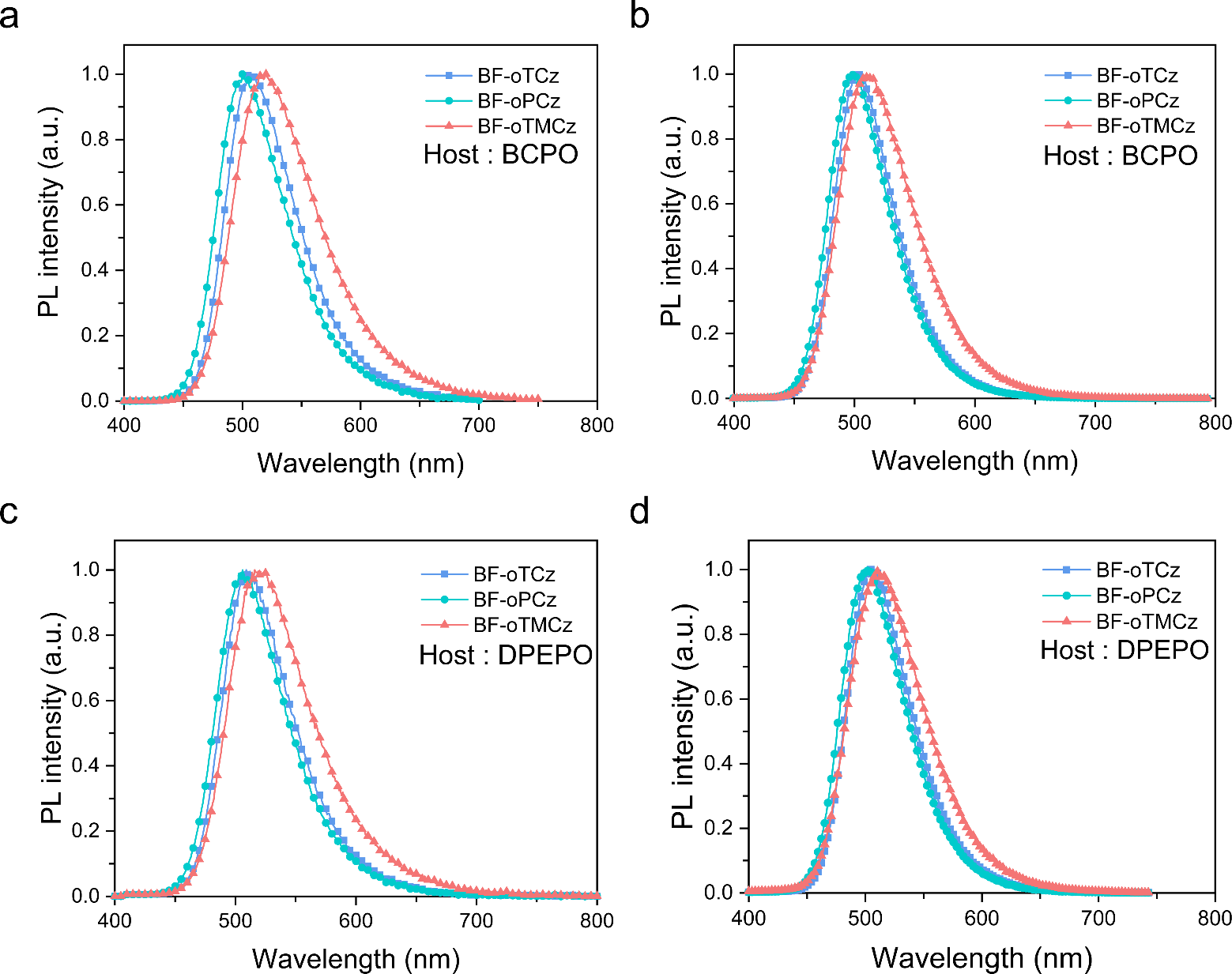


**Figure S5.** a) Steady-state PL spectra; b) Time-resolved PL spectra at 0-20 ns time range of BF-oTCz, BF-oPCz and BF-oTMCz with 20 wt% doped in BCPO at 300K. c) Steady-state PL spectra; d) Time-resolved PL spectra at 0-20 ns time range of BF-oTCz, BF-oPCz and BF-oTMCz with 20 wt% doped in DPEPO at 300K.


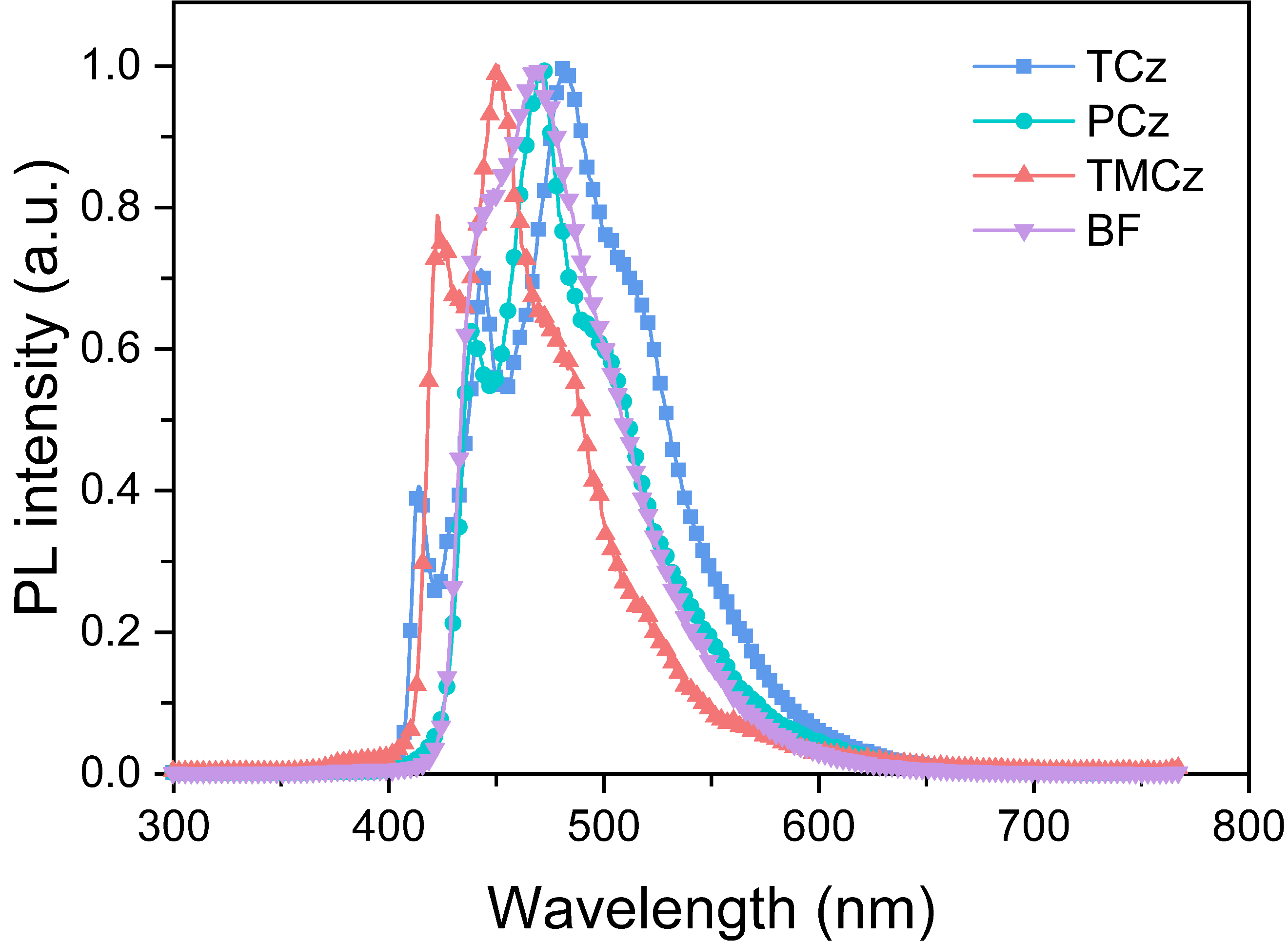


**Figure S6.** Phosphorescence spectra of TCz, PCz, TMCz and BF in dilute toluene solutions (10^−5^ M) at 77K.


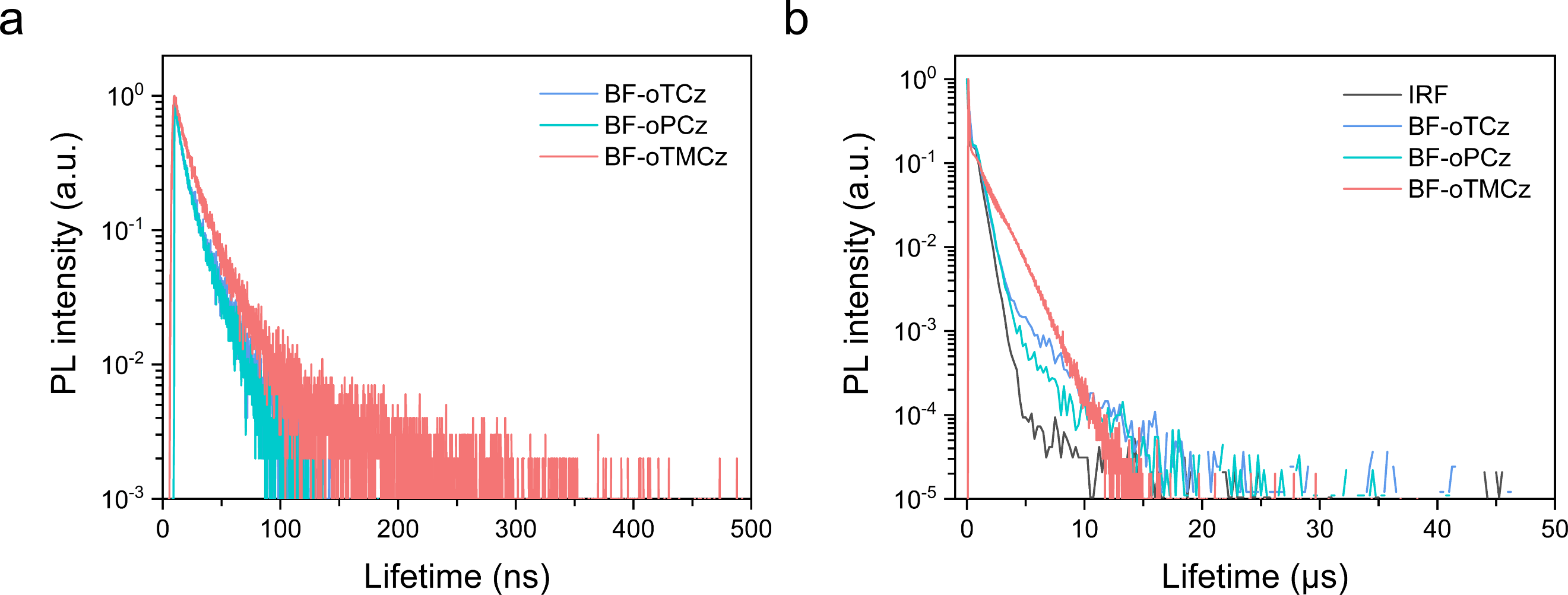


**Figure S7.** a). The comparison of transient PL decay curves of BF-oTCz, BF-oPCz and BF-oTMCz in dilute toluene solutions (10^−5^ M) at timescales of ns; b). the comparison of transient PL decay curves of BF-oTCz, BF-oPCz and BF-oTMCz in dilute toluene solutions (10^−5^ M) at timescales of μs.

**Table S5.** Photophysical data in dilute toluene solutions (10^−5^ M) (excited at 355 nm) of the investigated emitters.

| Emitter | λ_PL_^[a]^ | Φ_PL_/Φ_PF_/Φ_DF_^[b]^ | *τ*_PF_ /*τ*_DF_^[c]^ | *k*_ISC_/*k*_RISC_^[d]^ |
| --- | --- | --- | --- | --- |
|  | nm | % | ns/μs | 10^6^ s^-1^ |
| BF-oTCz | 500 | 78.3/-/- | 13.7/- | -/- |
| BF-oPCz | 496 | 73.5/-/- | 12.6/- | -/- |
| BF-oTMCz | 529 | 81.3/15.7/65.6 | 16.7/1.5 | 50.5/3.32 |

[a] the wavelength at PL maximum; [b] Φ_PL_, Φ_PF_ and Φ_DF_ are the overall PLQY, the quantum yields of prompt fluorescence and delayed fluorescence, respectively; [c] τ_PF_ and τ_DF_ are the lifetimes of prompt fluorescence and delayed fluorescence, respectively; [d] *k_IS_*_C_ and *k_RISC_* represent the rate constants of intersystem crossing (ISC) and reverse ISC (RISC).

Note: As shown in Figure **S7**, we observed almost no TADF emission from BF-oTCz and BF-oPCz. We speculate that the quite long-lived TADF emission of BF-oTCz and BF-oPCz might have been quenched by trace dissolved oxygen which is impossible to remove completely in our sample cell, even though we continuously bubbled Ar for over 20 minutes before PL measurements.


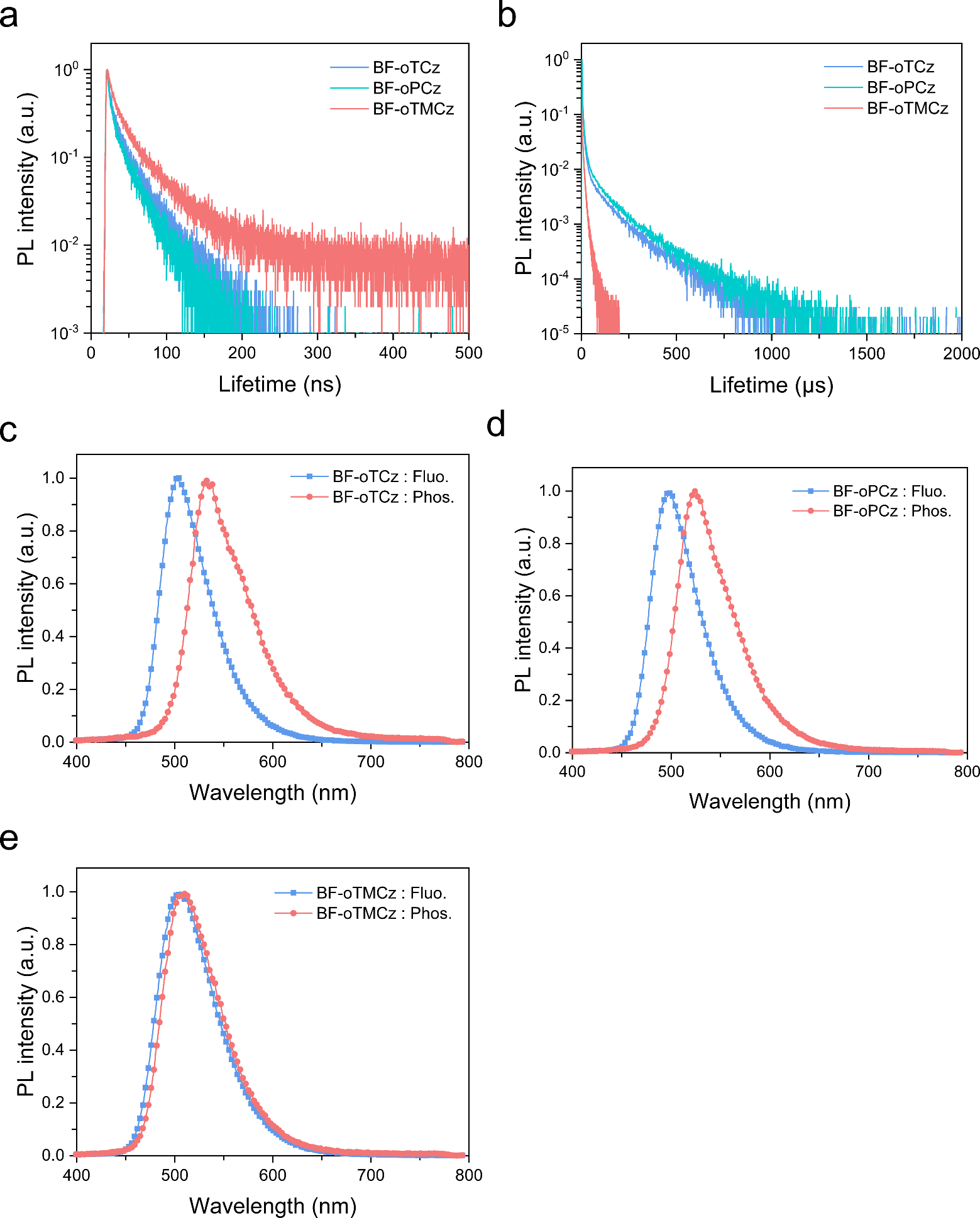


**Figure S8.** a-b) Transient PL decay curves of BF-oTCz, BF-oPCz and BF-oTMCz in 20 wt% doped DPEPO films; c-e) time-resolved PL spectra of BF-oTCz, BF-oPCz and BF-oTMCz in 20 wt% doped DPEPO films at 77 K. Fluo.: fluorescence; Phos.: phosphorescence;

**Table S6.** Photophysical data in 20 wt%-doped DPEPO films (excited at 355 nm) of the investigated emitters.

| Emitter | λ_PL_^[a]^ | Φ_PL_/Φ_PF_/Φ_DF_^[b]^ | *τ*_PF_ /*τ*_DF_^[c]^ | *k*_ISC_/*k*_RISC_^[d]^ | E_S_/E_T_/ΔE_ST_^[e]^ |
| --- | --- | --- | --- | --- | --- |
|  | nm | % | ns/μs | 10^6^ s^-1^ | eV |
| BF-oTCz | 508 | 96.1/81.8/14.3 | 22.0/147.1 | 8.26/0.06 | 2.65/2.50/0.15 |
| BF-oPCz | 503 | 88.2/74.4/13.8 | 18.3/125.3 | 16.9/0.05 | 2.68/2.54/0.14 |
| BF-oTMCz | 515 | 93.2/5.7/87.5 | 31.3/7.1 | 30.0/2.27 | 2.65/2.62/0.03 |

[a] the wavelength at PL maximum; [b] Φ_PL_, Φ_PF_ and Φ_DF_ are the overall PLQY, the quantum yields of prompt fluorescence and delayed fluorescence, respectively; [c] τ_PF_ and τ_DF_ are the lifetimes of prompt fluorescence and delayed fluorescence, respectively; [d] *k_IS_*_C_ and *k_RISC_* represent the rate constants of intersystem crossing (ISC) and reverse ISC (RISC); [e] energy levels of S_1_ and T_1_ were estimated from fluorescence and phosphorescence spectra at 77 K; energy gaps between lowest singlet and triplet excited states (ΔE_ST_) were obtained from E_S_-E_T_.


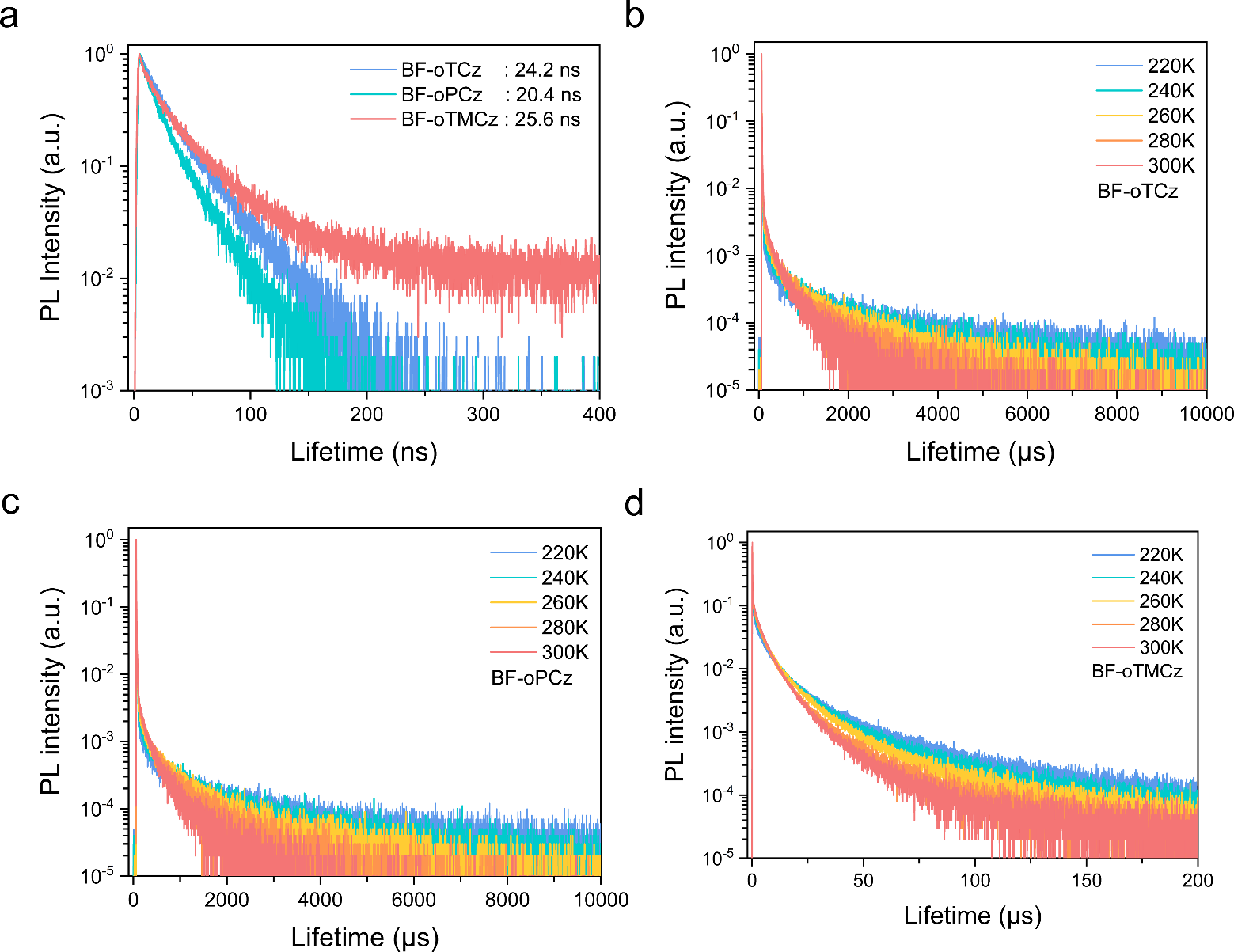


**Figure S9.** Transient decay spectra of BF-oTCz, BF-oPCz and BF-oTMCz with 20 wt% doped in BCPO. a) 0-400 ns time range at 300K, excited at 355 nm; b-d) at different temperatures (from 300 to 220 K, excited at 355 nm).

Rate constants of the investigated compounds in 20 wt% BCPO films at room temperature were determined from the obtained quantum yields and lifetimes of the prompt fluorescence (PF) and delayed fluorescence (DF) components according to equations S1-S4, which were derived by Masui and Adachi et al.

k_r_ = Φ_PF_k_PF_ Equation S1

k_ISC_ = (1-Φ_PF_) k_PF_ Equation S2

k_RISC_ = (k_PF_k_DF_/k_ISC_) (Φ_DF_/Φ_PF_) Equation S3

k_nr_ = k_DF_ - Φ_PF_k_RISC_  Equation S4

**6.** **Thermogravimetric Analysis (TGA) and Differential Scanning Calorimetry (DSC)**

**
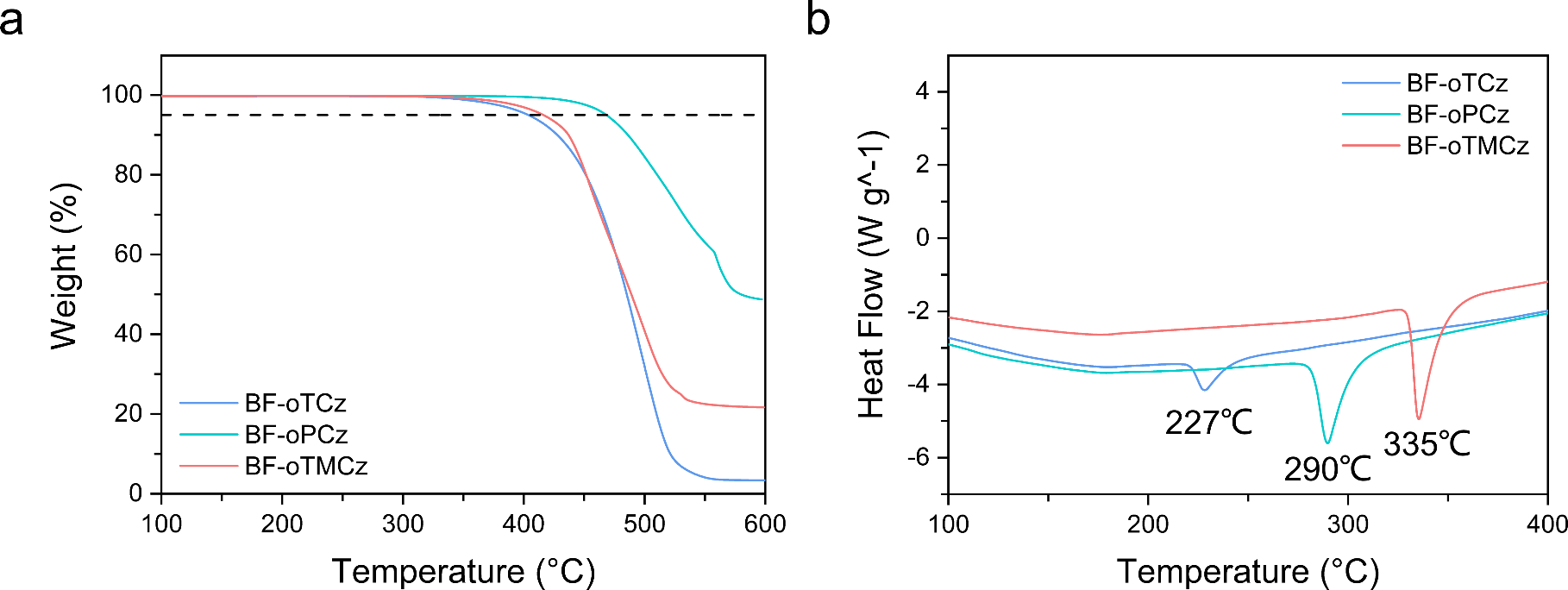
**

**Figure S10.** a) TGA curves of BF-oTCz, BF-oPCz and BF-oTMCz. The black dashed line marks 95% of the original sample weight. b) DSC curves of BF-oTCz, BF-oPCz and BF-oTMCz.

**7. Cyclic Voltammetry**


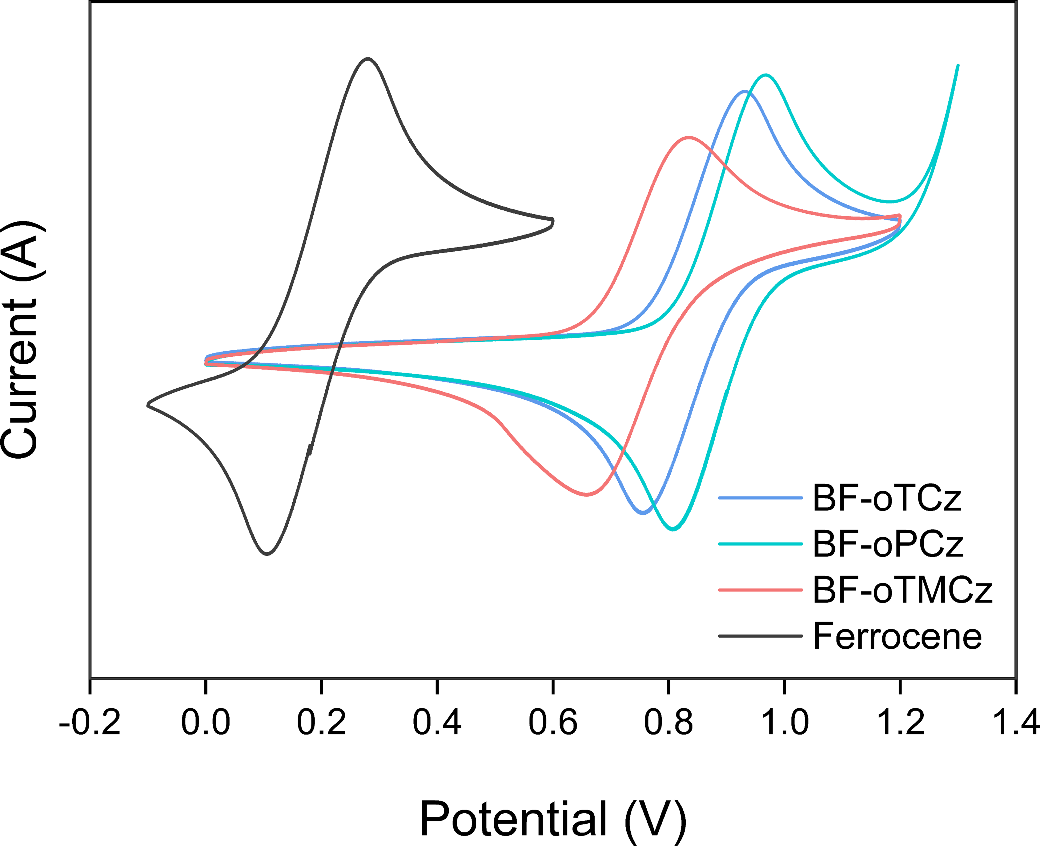


**Figure S11.** Cyclic voltammograms for the oxidation of BF-oTCz, BF-oPCz and BF-oTMCz in dichloromethane at room temperature (ferrocenium/ferrocene couple was used as an internal standard).

Cyclic voltammetry was performed at room temperature in anhydrous and argon-saturated dichloromethane solutions of 0.1 M tetrabutylammonium hexafluorophosphate and 1.0 mmol investigated compounds with a CHI840D electrochemical analyzer. Glassy carbon, platinum wire and Ag/Ag+ (0.01 M of AgNO3 in acetonitrile) were selected as the working electrode, auxiliary electrode and reference electrode, respectively. The ferrocenium/ferrocene couple was used as an internal standard. The HOMO and LUMO energy levels were estimated from the cyclic voltammetry and optical bandgaps (E_g_) determined from the onset of the absorption band (λ_onset_).

E_HOMO_ = - [E_ox_ - E_Fc/Fc+_ + 4.8] eV;

E_g_ = 1241/ λ_onset_;

E_LUMO_ = E_HOMO_ + E_g_

Based on the above formulas, HOMO/LUMO energy levels of BF-oTCz, BF-oPCz and BF-oTMCz were calculated to be -5.37/2.75 eV, 5.42/2.75 eV and -5.28/-2.75 eV, respectively.

**Table S7.** Summary of CV data and energy levels.

| Compound | E_ox_^[a]^ | E_Fc/Fc+_^[b]^ | E_g_^[c]^ | E_HOMO_^[d]^ | E_LUMO_^[e]^ |
| --- | --- | --- | --- | --- | --- |
|  | [eV] | [eV] | [eV] | [eV] | [eV] |
| BF-oTCz | 0.767 | 0.193 | 2.62 | -5.37 | 2.75 |
| BF-oPCz | 0.814 | 0.193 | 2.67 | -5.42 | -2.75 |
| BF-oTMCz | 0.670 | 0.193 | 2.53 | -5.28 | -2.75 |

[a] The oxidation potentials (E_OX_) were acquired from the onset of first oxidation potentials in cyclic voltammograms (see Figure S15); [b] ferrocenium/ferrocene couple was used as an internal standard; [c] calculated from the absorption edge λ_onset_ (see Figure S5) using equation: 1241/λ_onset_; [d] calculated using the equation: E_HOMO_ = - [E_OX_ - E_Fc_/_Fc+_ + 4.8] eV; e calculated from E_g_ and E_HOMO_ using the equation: E_LUMO_ = (E_HOMO_ + E_g_) (eV).

**8.** **Device Fabrication and Characterization**

Indium tin oxide (ITO) glass substrates were cleaned successively in an ultrasonic bath containing deionized water, acetone and ethanol. They were then blown dry with N_2_ and treated in UV-ozone ambient conditions for 15 min prior to film deposition. Under high vacuum (< 5 × 10^−5^ Pa), the organic materials were deposited onto the ITO glass substrates by vacuum thermal evaporation in an inert chamber at a rate of 1 Å s^-1^. Al layer (100 nm) were deposited by vacuum thermal evaporation in another inert chamber at a base pressure less than 9 × 10^−5^ Pa. The intersection of the ITO and the Al electrodes gave an active device area of 9 mm^-2^. EL measurements were carried out at room temperature under ambient conditions. The EL spectra, CIE coordinates, CEs, PEs, EQEs, and current density-voltage-luminance curves (J-V-L) of the OLEDs were measured by an integrated optoelectronic performance test system including a calibrated spectra radiometer (TOPCON SR-UL1R) and a Keithley 2400 source meter.

The intrinsic carrier-transporting properties of these compounds are examined by fabricating hole‐only devices (HOD) and electron‐only devices (EOD) (**Figure S11**). According to space-charge limited current (SCLC) model, the hole and electron mobilities were estimated to be 18.18 × 10^-6^ and 3.12 × 10^-6^ cm^2^ V^−1^ s^−1^ for BF-oTCz, 13.78 × 10^-6^ and 4.71 × 10^-6^ cm^2^ V^−1^ s^−1^ for BF-oPCz and 51.98 × 10^-6^ and 4.31 × 10^-6^ cm^2^ V^−1^ s^−1^ for BF-oTMCz, respectively, revealing their relatively balanced bipolar charge-transporting characters.


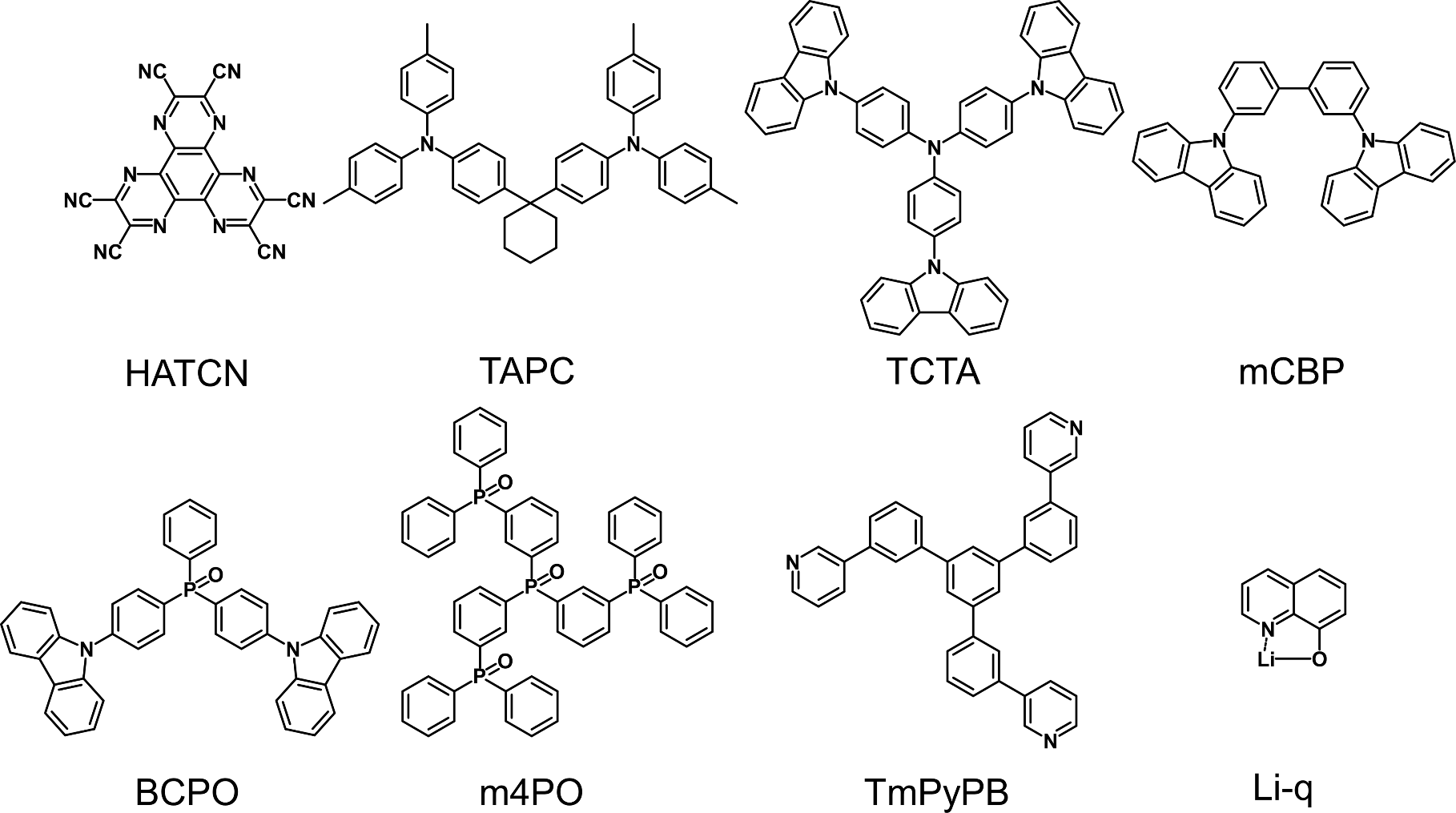


**Figure S12.** Chemical structures of the functional materials used in the OLEDs.


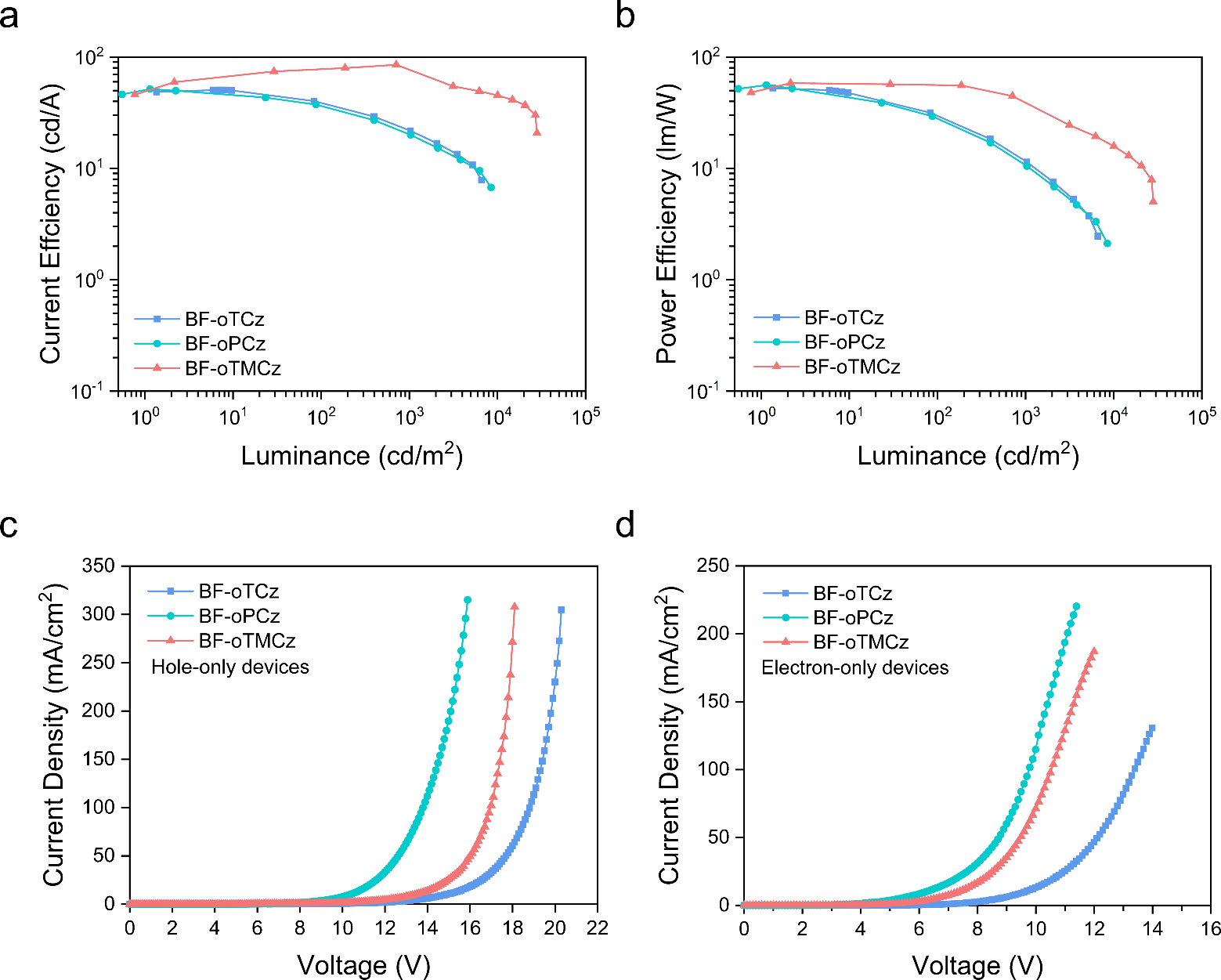


**Figure S13.** OLEDs based on BF-oTCz, BF-oPCz and BF-oTMCz with 20 wt% doped in BCPO. a) Current Efficiency (CE) versus luminance characteristics; b) Power Efficiency (PE) versus luminance characteristics; c) The current density versus voltage curves of the hole‐only devices (HOD); d) The current density versus voltage curves of the electron‐only devices(EOD).


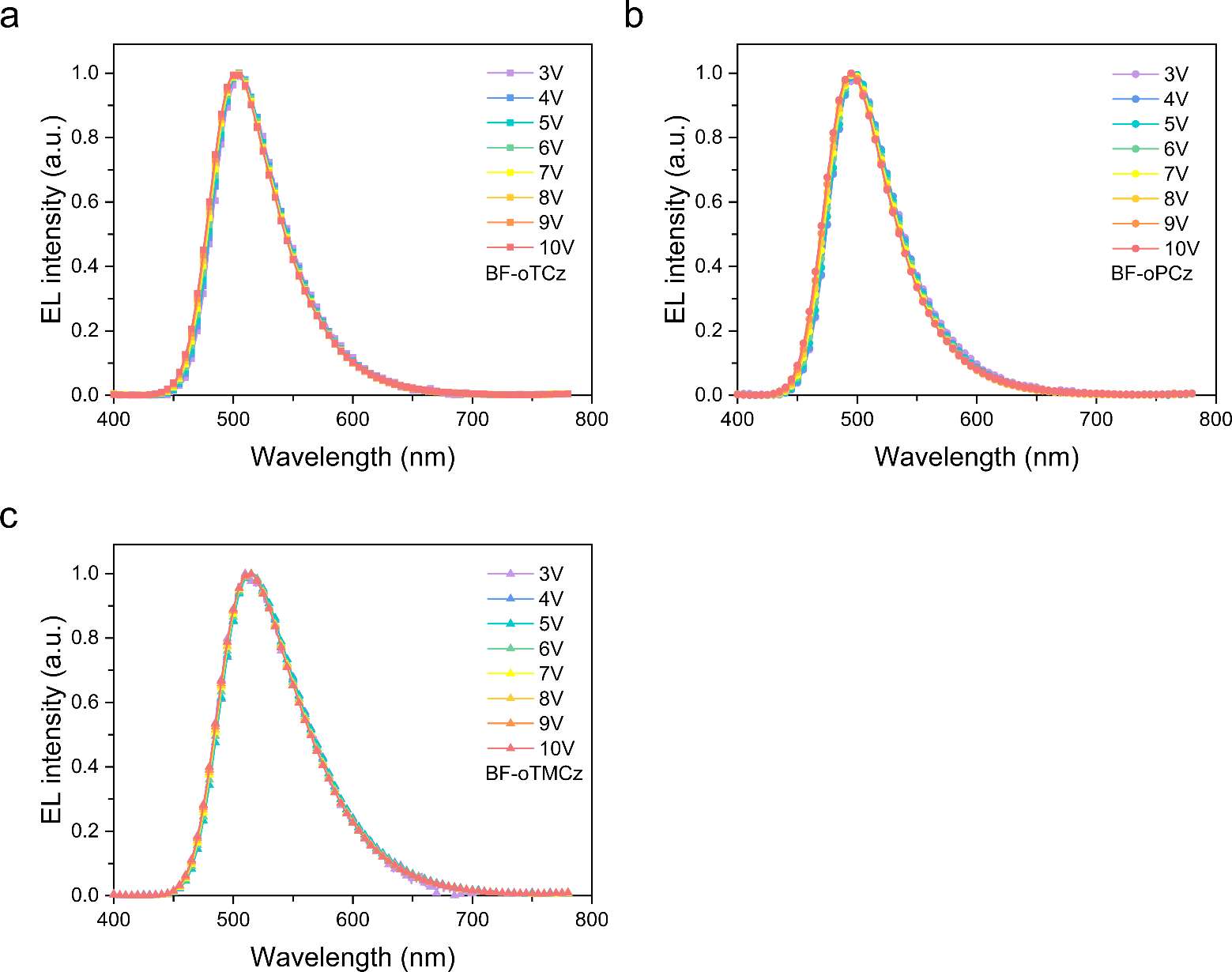


**Figure S14.** EL spectra at various voltages(3-10V), based on BF-oTCz, BF-oPCz and BF-oTMCz with 20 wt% doped in BCPO.


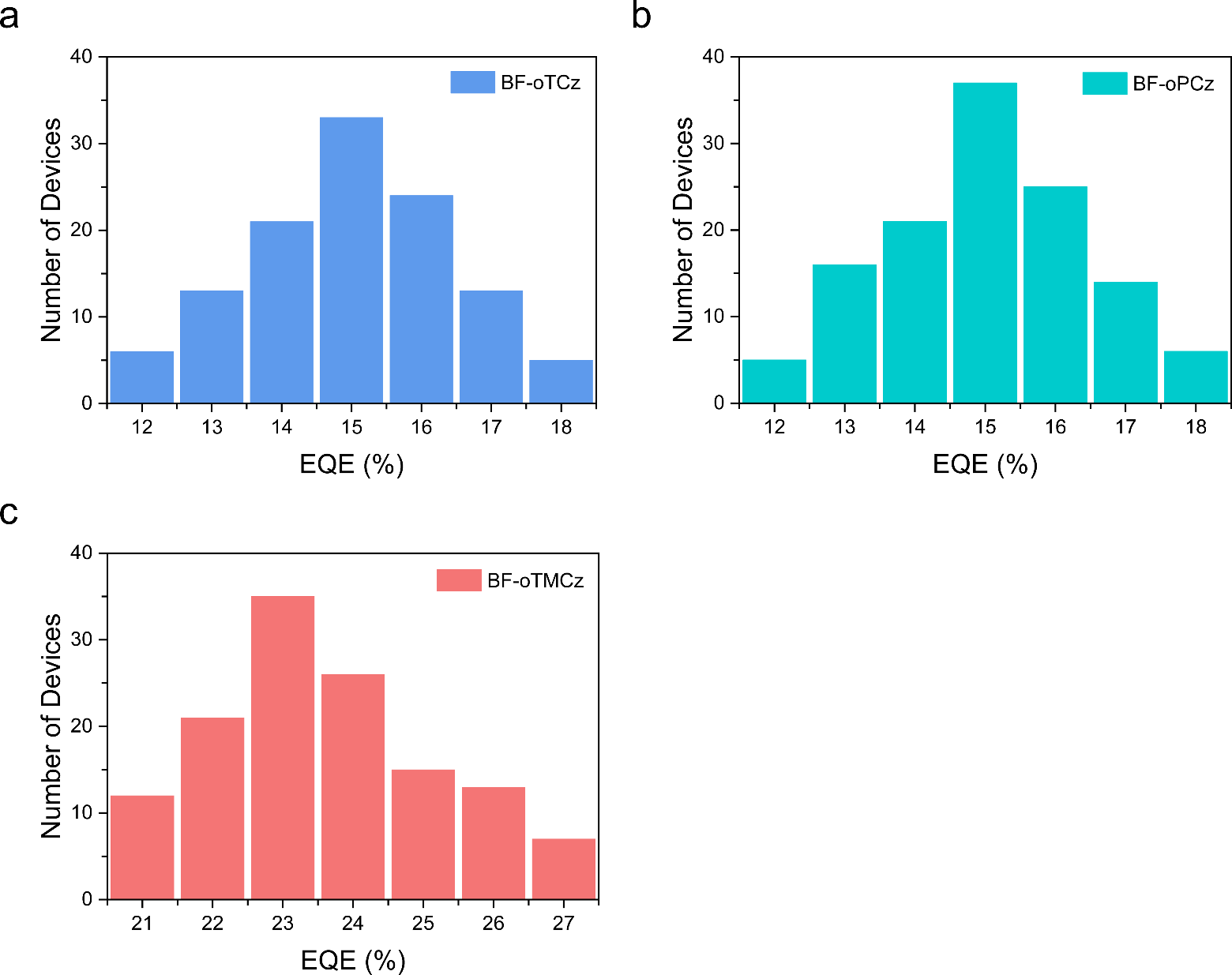


**Figure S15.** a-c) Statistical histograms of the peak EQEs for the devices based on BF-oTCz, BF-oPCz and BF-oTMCz.


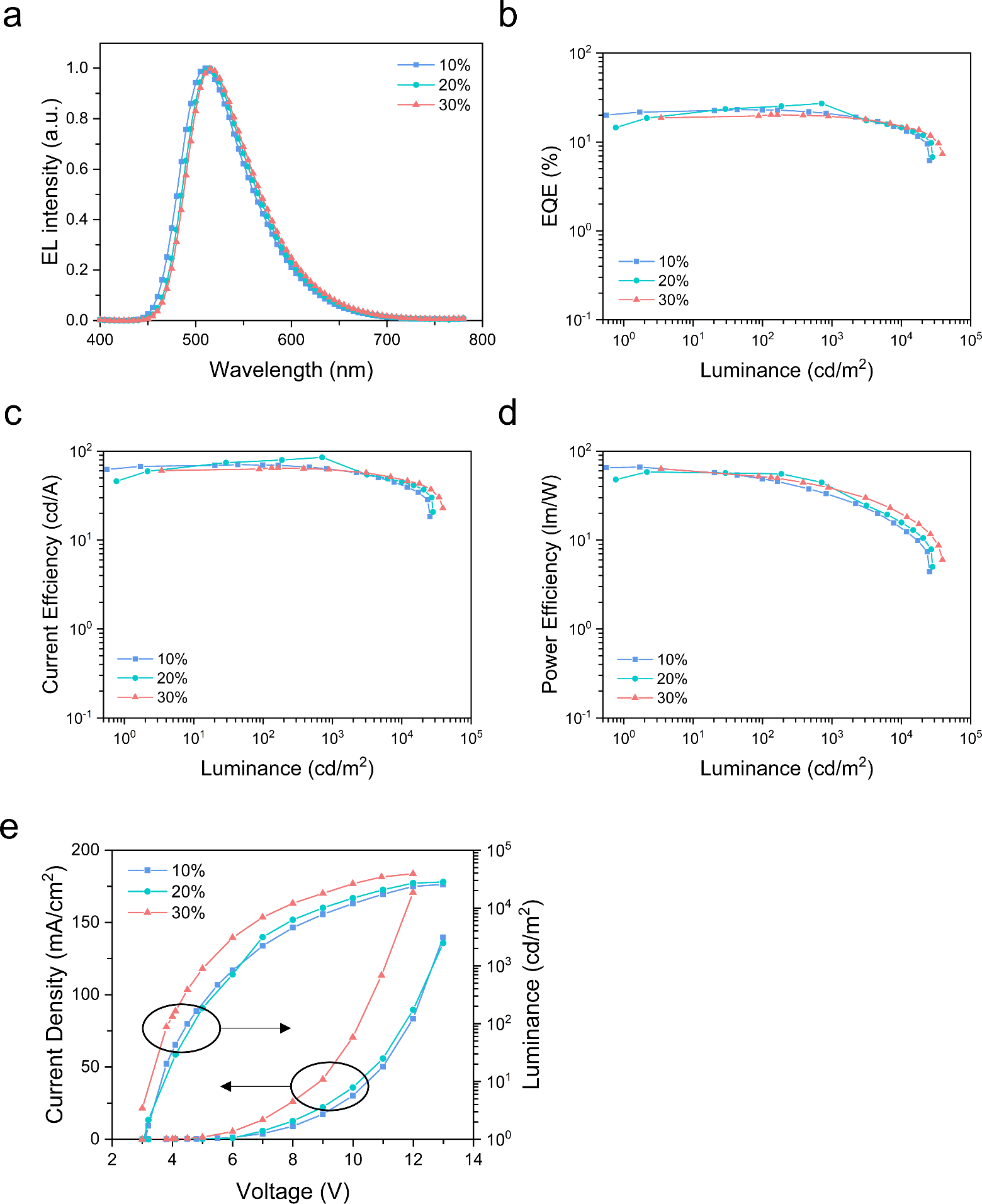


**Figure S16.** OLEDs based on BF-oTMCz with 10 wt%, 20 wt% and 30 wt% doped in BCPO. a) Electroluminescence (EL) spectra at 6V; b) External quantum efficiency (EQE) versus luminance characteristics; c) Current Efficiency (CE) versus luminance characteristics; d) Power Efficiency (PE) versus luminance characteristics; e) Current Density(J)-voltage(V)- luminance(L) characteristics.

**Table S8.** The performance of the electroluminescence (EL) devices of BF-oTMCz in 10/20/30 wt% doped in BCPO.

| Concentrations | λ_EL_^[a]^ | L_max_^[b]^ | EQE_max_/EQE_1000_^[c]^ | CE_max_^[d]^ | PE_max_^[e]^ | CIE^[f]^ |
| --- | --- | --- | --- | --- | --- | --- |
| wt% | nm | cd/m^2^ | % | cd/A | lm/W | (x, y) |
| 10 | 510 | 25,530 | 23.1/20.8 | 70.6 | 66.3 | (0.2682, 0.5484) |
| 20 | 515 | 28,150 | 27.1/26.0 | 85.2 | 58.3 | (0.2851, 0.5715) |
| 30 | 516 | 39,140 | 20.3/19.4 | 64.8 | 63.4 | (0.2947, 0.5777) |

[a] Emission peak of electroluminescence at 6V; [b] Maximum luminance; [c] Maximum external quantum efficiencies (EQE); EQE at 100/1000/10000 cd m^-2^; [d] Maximum current efficiency; [e] Maximum power efficiency; [f] Commission Internatinale de L’Eclairage coordinates measured at 6V.

**9.** **NMR Spectra**


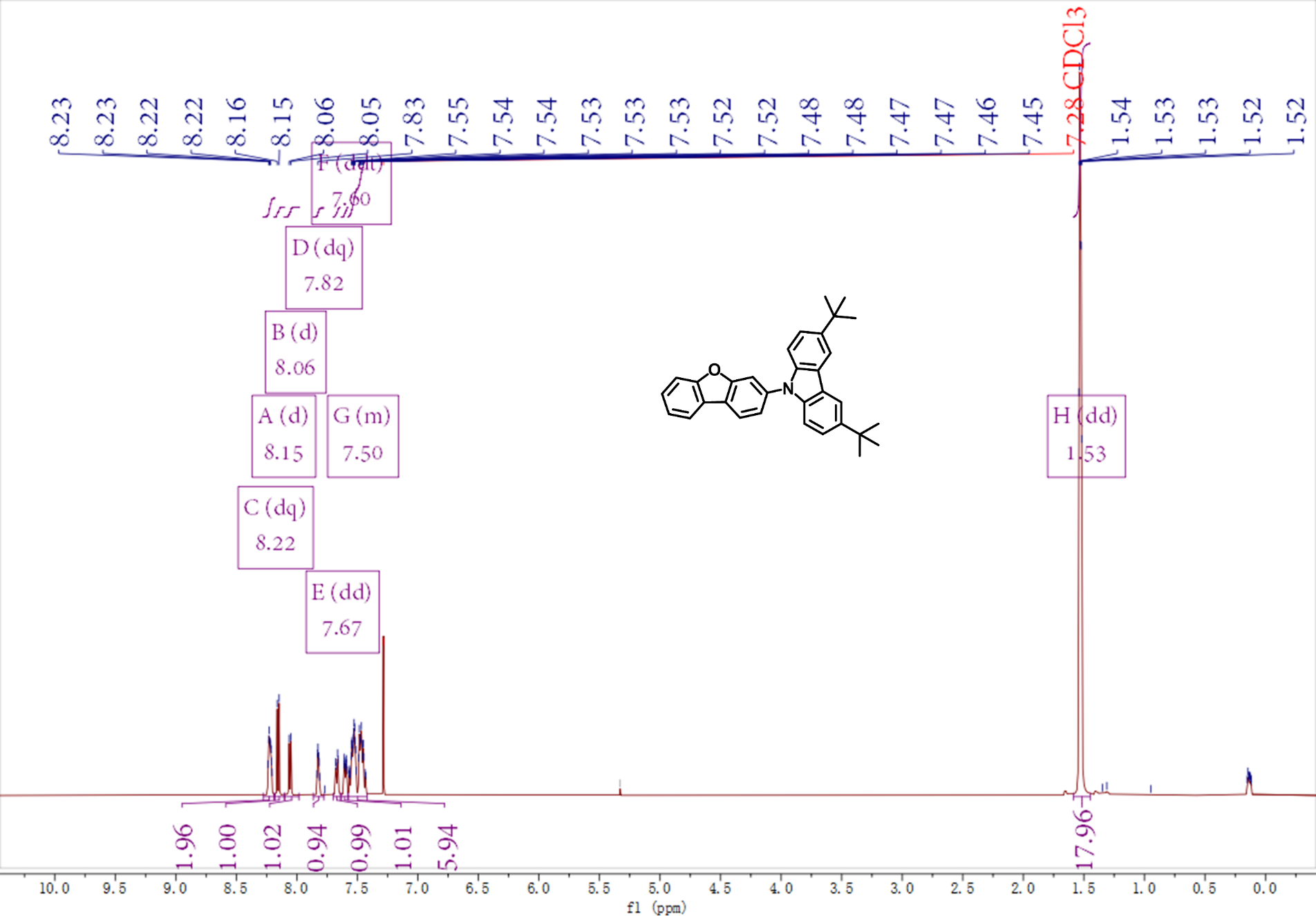


**Figure S17.** ^1^H-NMR spectrum of F-oTCz (500 MHz, CDCl3)


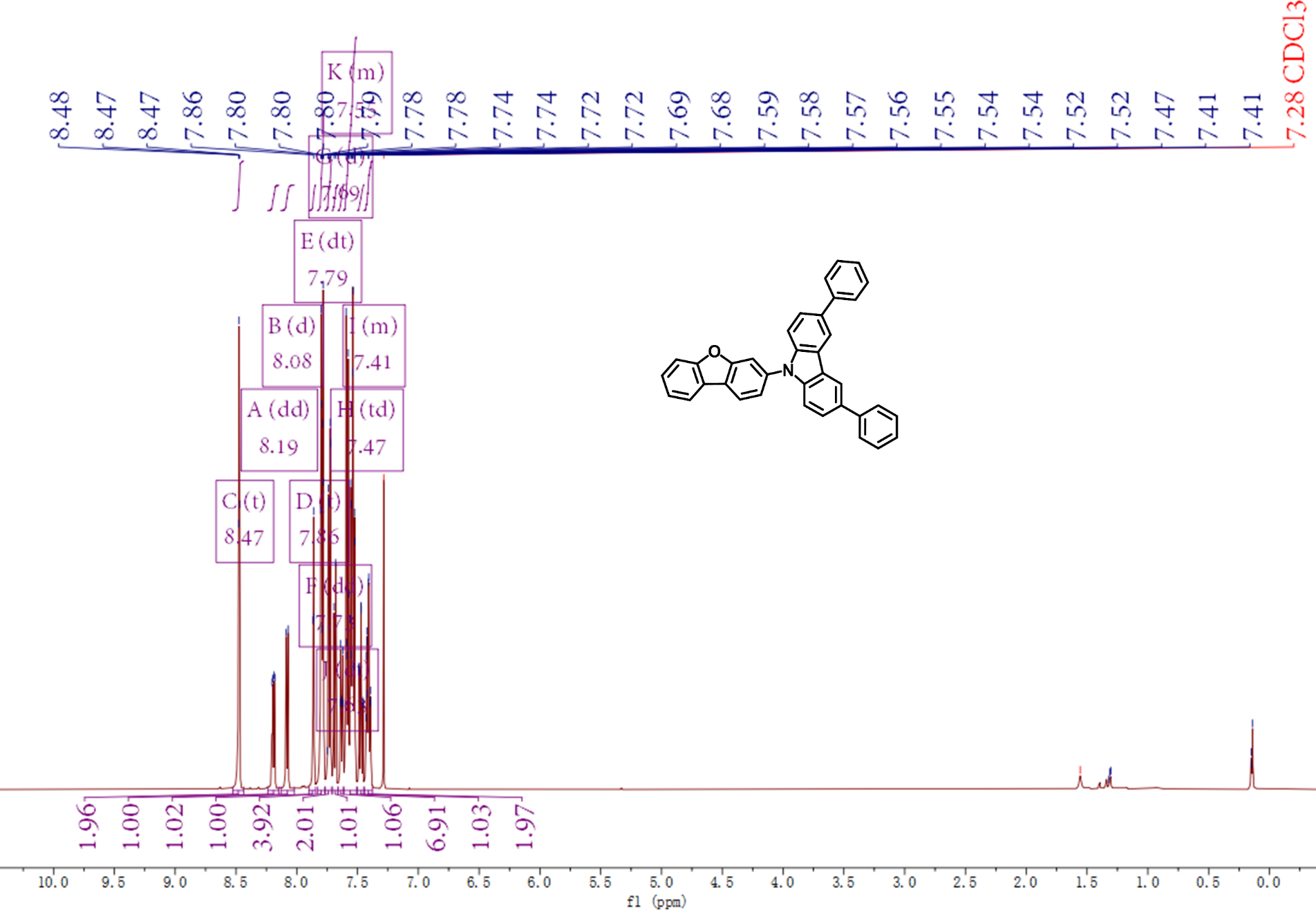


**Figure S18.** ^1^H-NMR spectrum of F-oPCz (500 MHz, CDCl3)


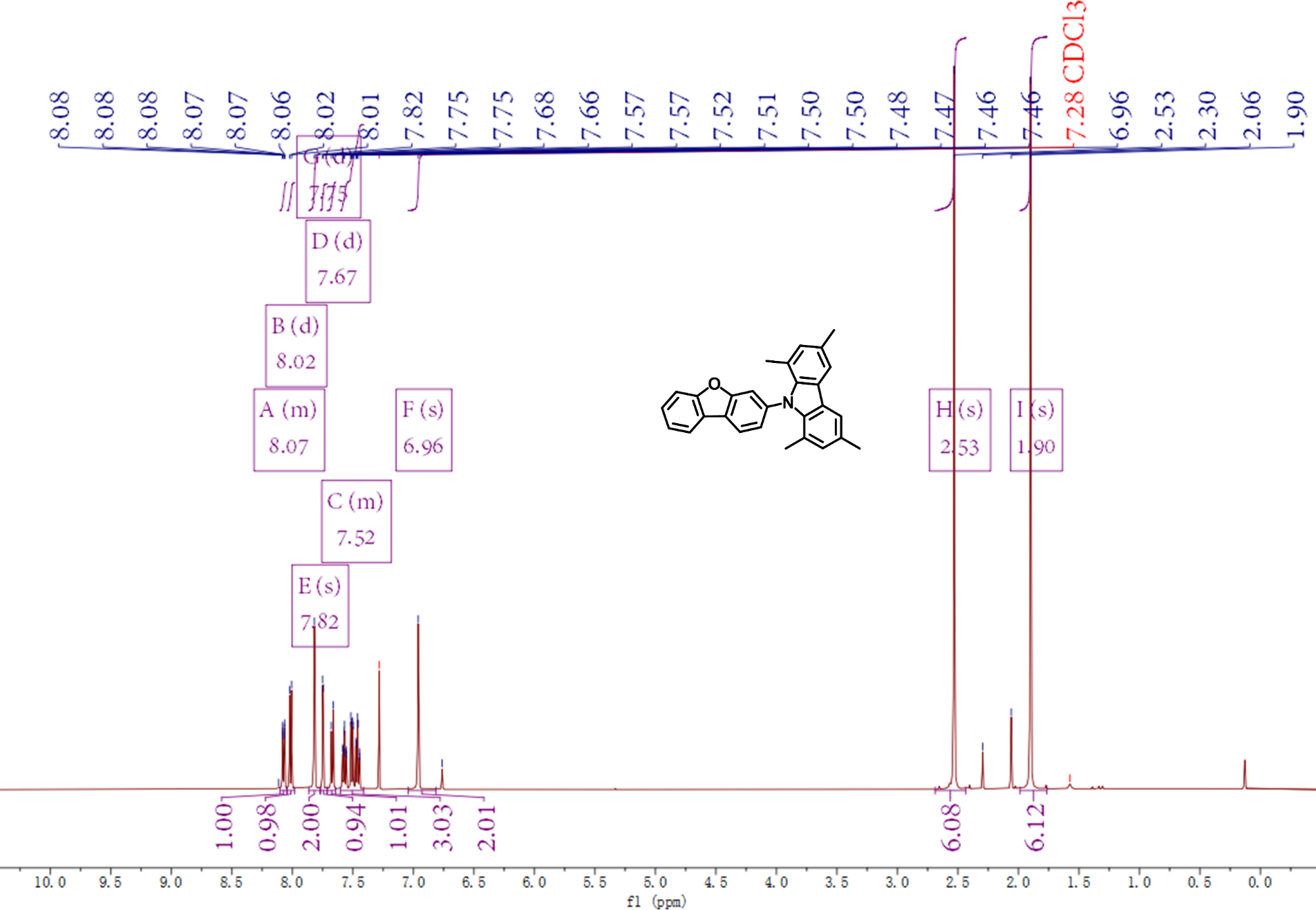


**Figure S19.** ^1^H-NMR spectrum of F-oTMCz (500 MHz, CDCl3)


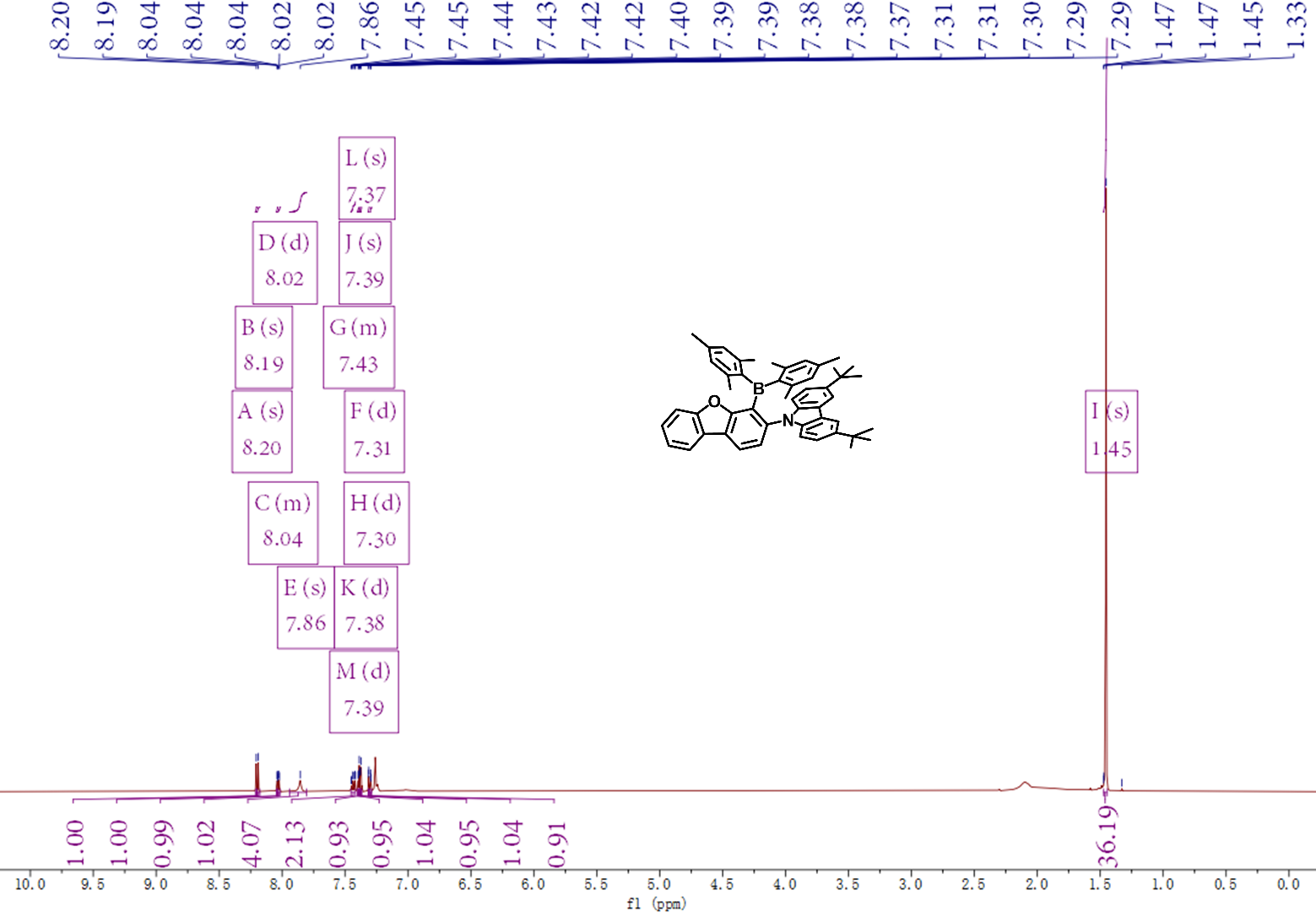


**Figure S20.** ^1^H-NMR spectrum of BF-oTCz (500 MHz, CDCl3)


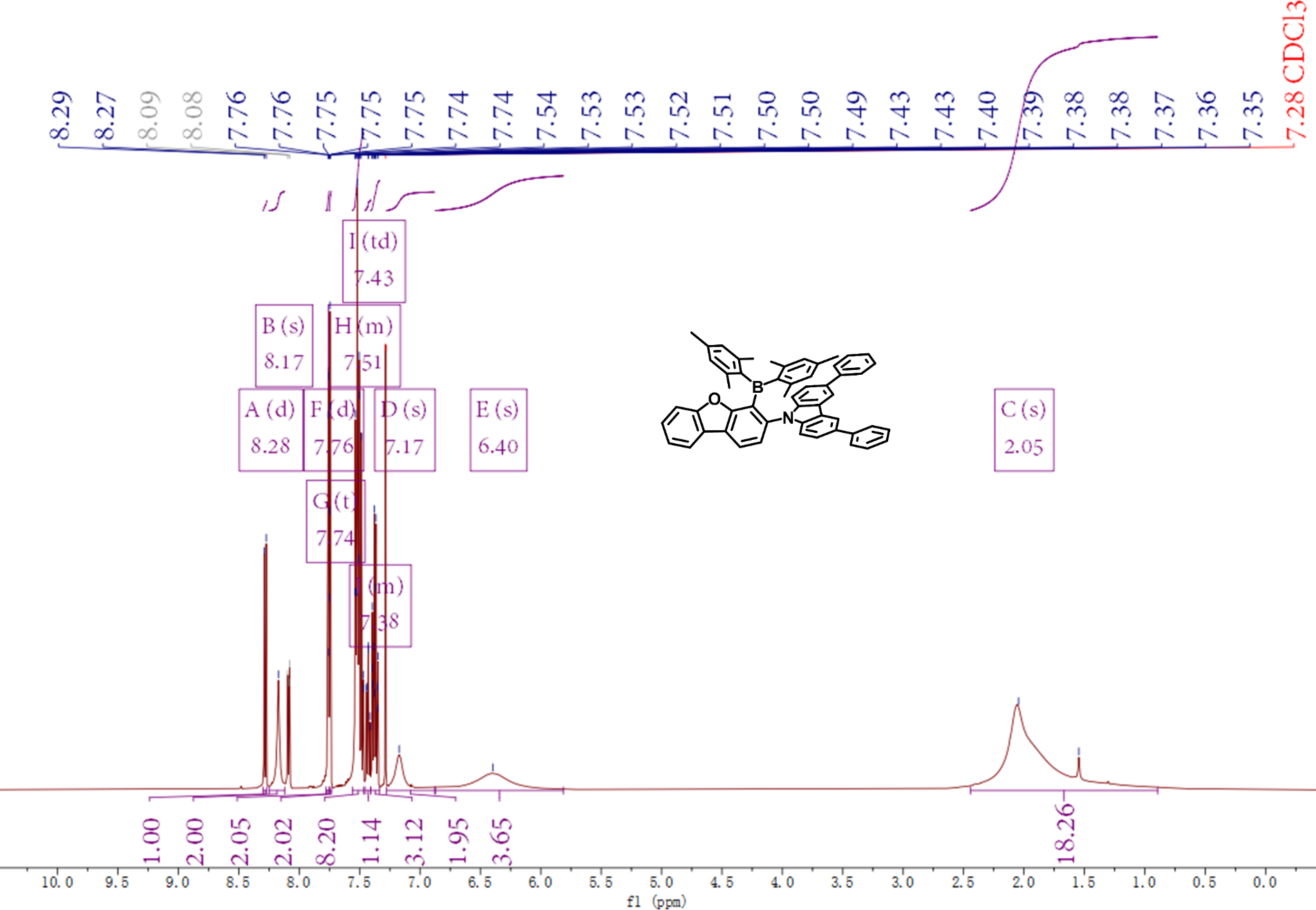


**Figure S21.** ^1^H-NMR spectrum of BF-oPCz (500 MHz, CDCl3)


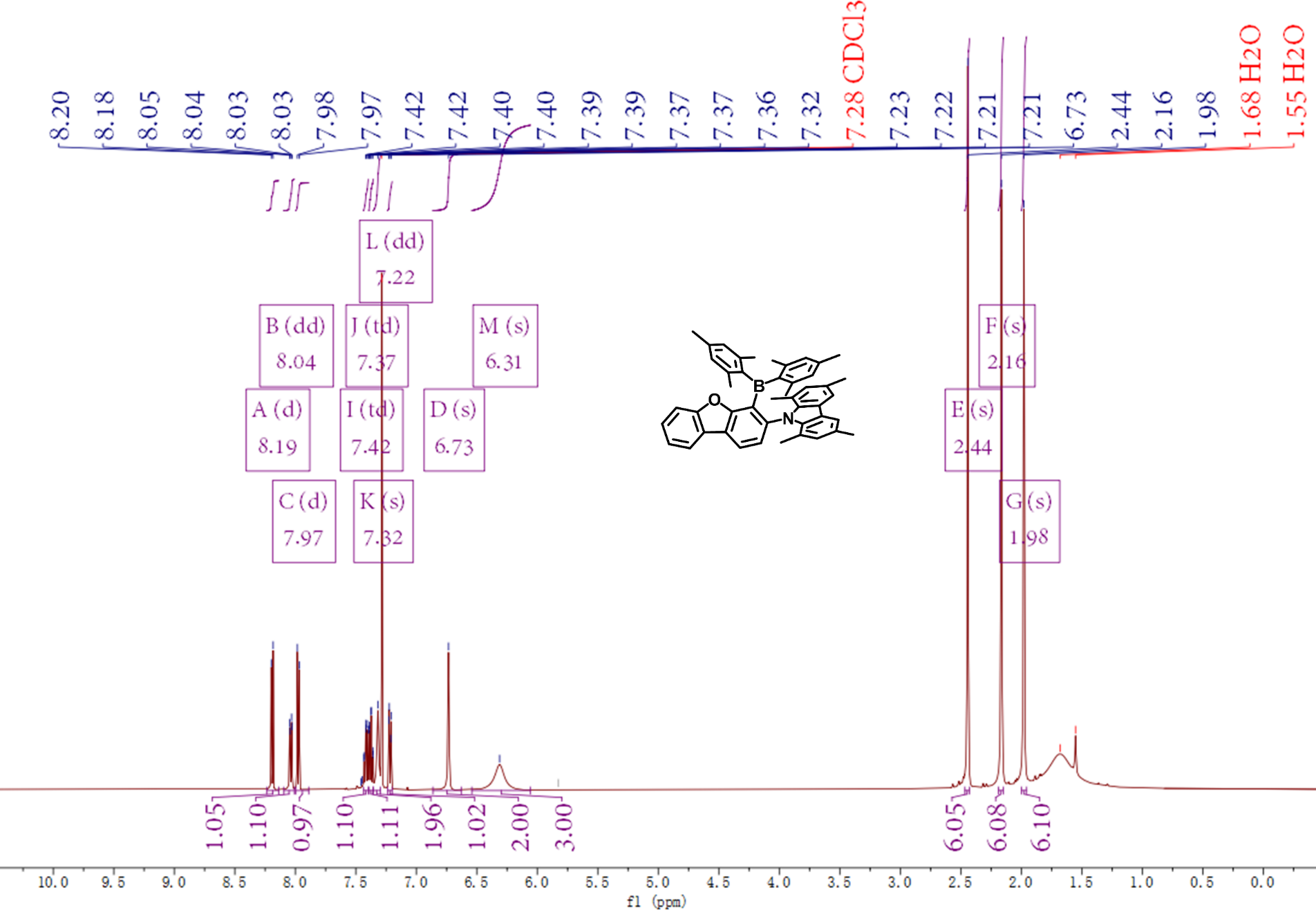


**Figure S22.** ^1^H-NMR spectrum of BF-oTMCz (500 MHz, CDCl3)


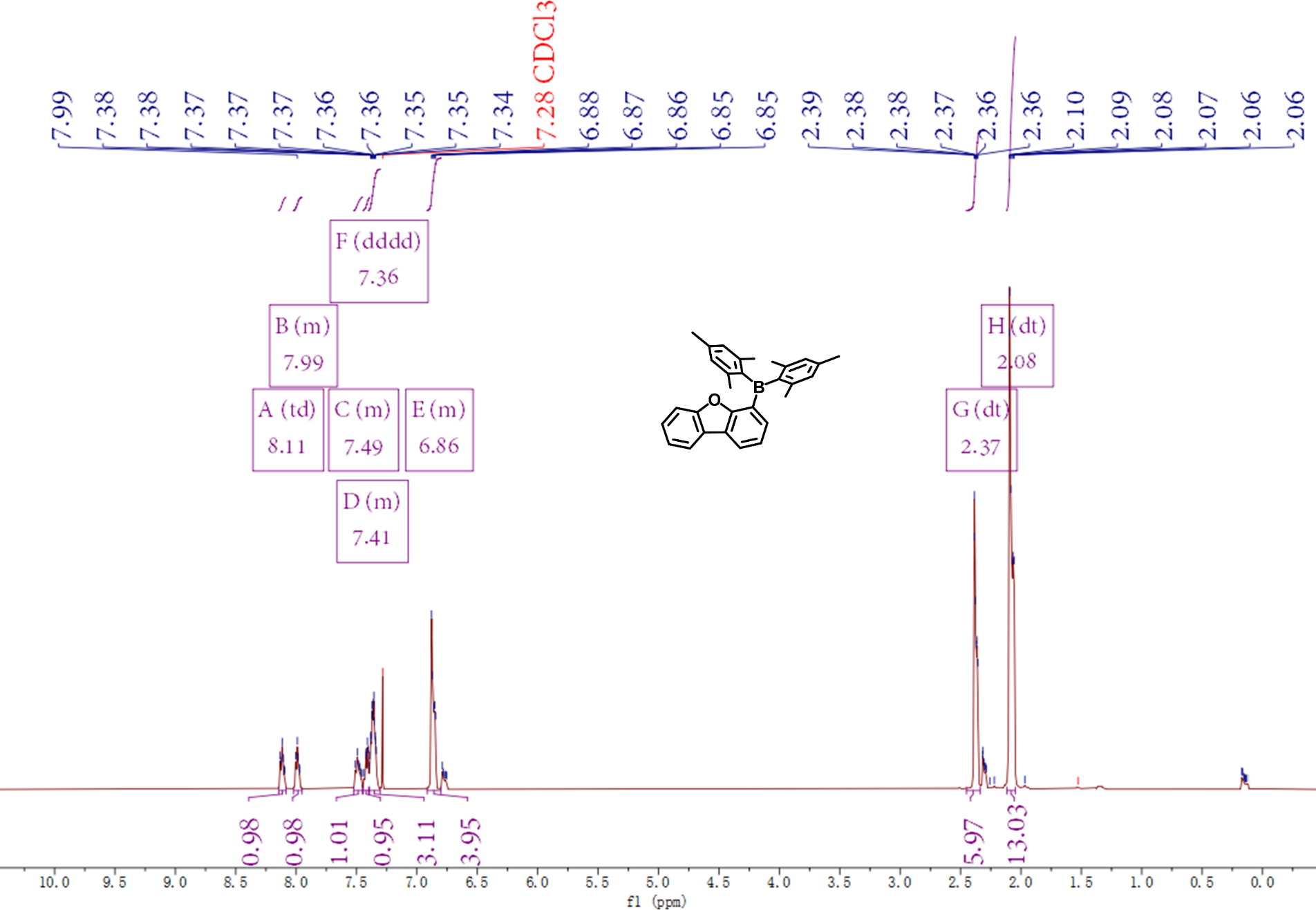


**Figure S23.** ^1^H-NMR spectrum of BF (500 MHz, CDCl3)


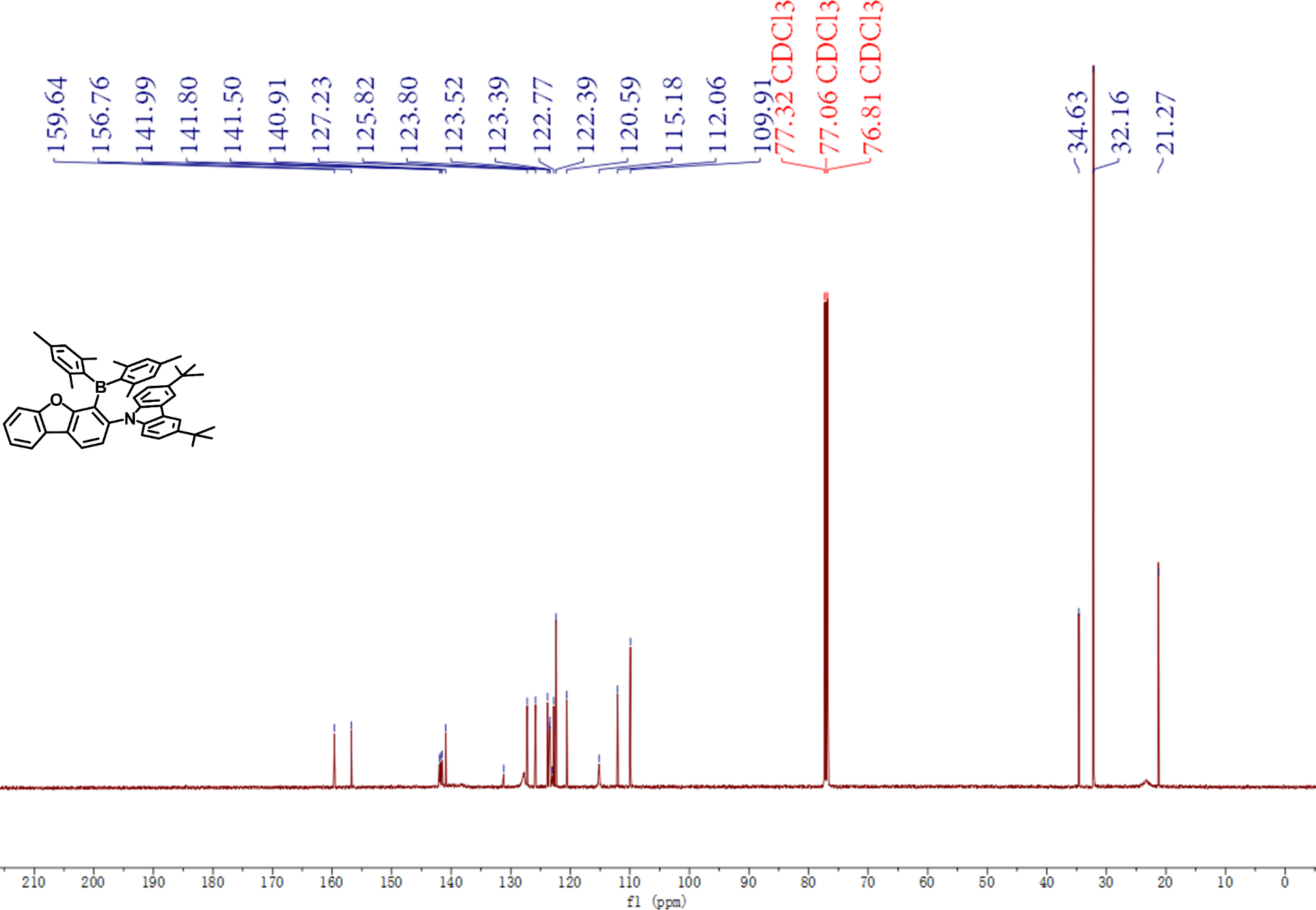


**Figure S24.** ^1^ C-NMR spectrum of BF-TCz (500 MHz, CDCl3)


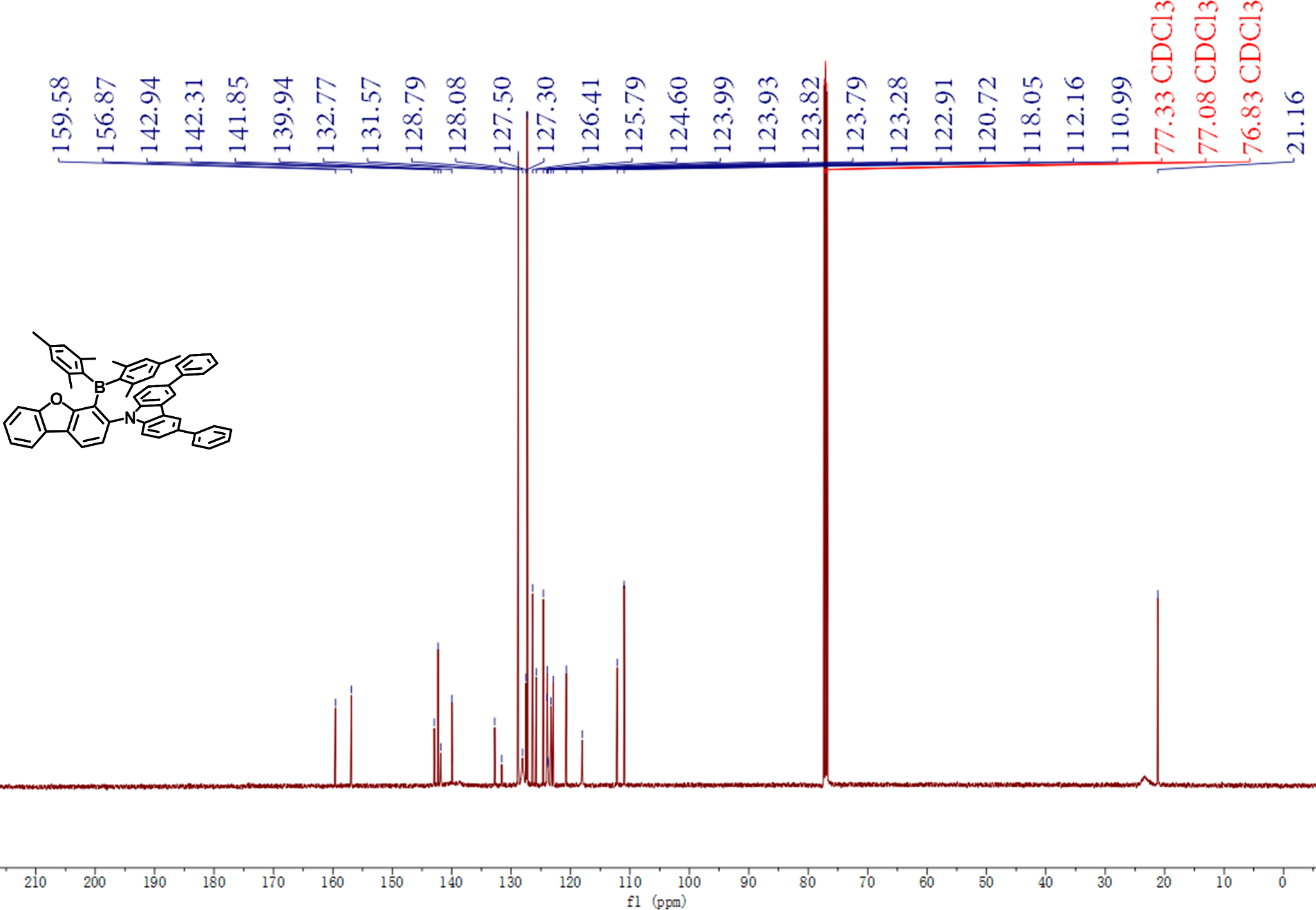


**Figure S25.** ^1^ C-NMR spectrum of BF-PCz (500 MHz, CDCl3)


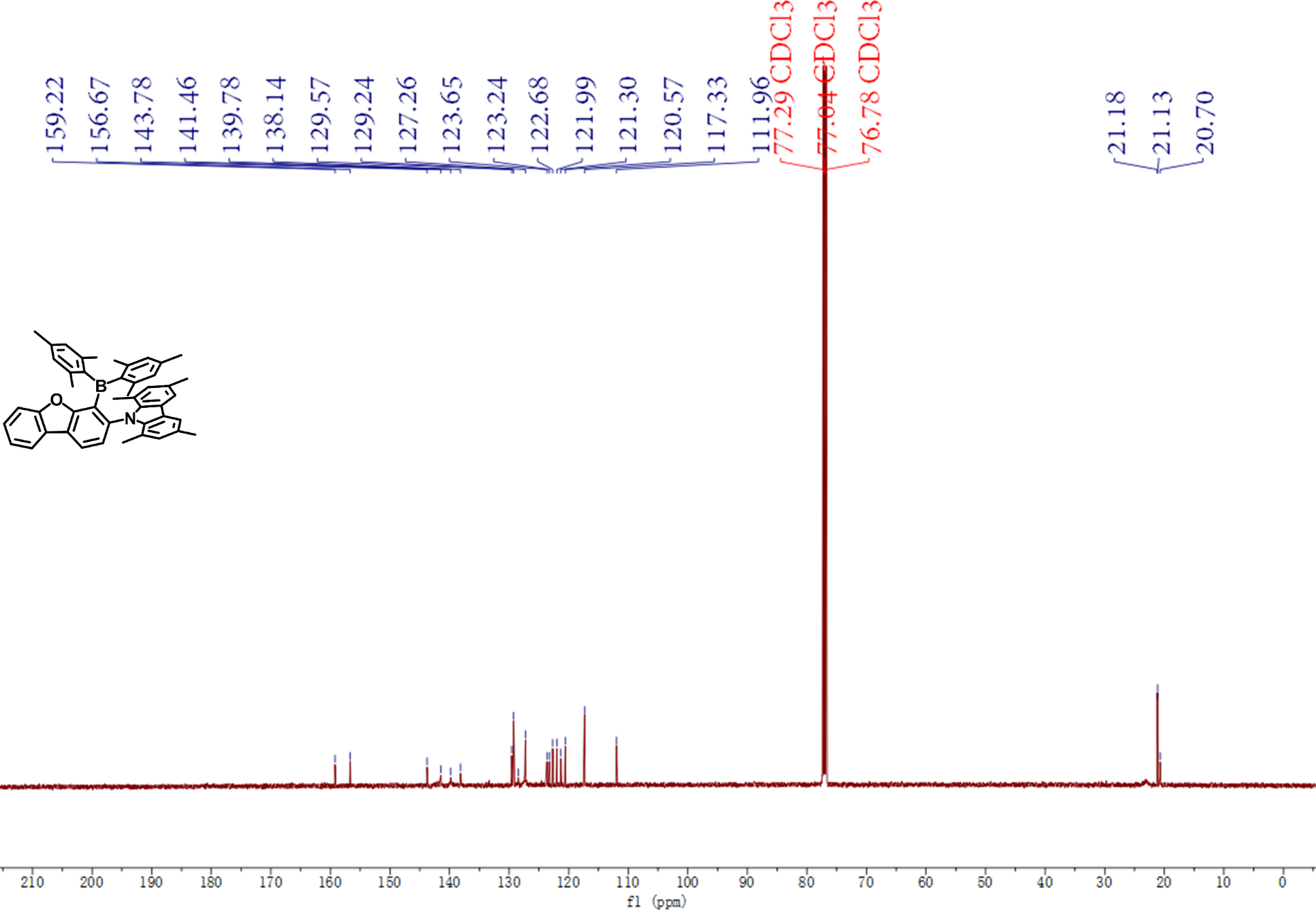


**Figure S26.** ^1^ C-NMR spectrum of BF-TMCz (500 MHz, CDCl3)
